# Cancer-Drug Associations: A Complex System

**DOI:** 10.1371/journal.pone.0010031

**Published:** 2010-04-02

**Authors:** Ertugrul Dalkic, Xuewei Wang, Neil Wright, Christina Chan

**Affiliations:** 1 Center for Systems Biology, Michigan State University, East Lansing, Michigan, United States of America; 2 Cellular and Molecular Biology Lab, Department of Chemical Engineering and Materials Science, Michigan State University, East Lansing, Michigan, United States of America; 3 Cell and Molecular Biology Program, Michigan State University, East Lansing, Michigan, United States of America; 4 Department of Chemical Engineering and Materials Science, Michigan State University, East Lansing, Michigan, United States of America; 5 Department of Mechanical Engineering, Michigan State University, East Lansing, Michigan, United States of America; 6 Department of Biochemistry and Molecular Biology, Michigan State University, East Lansing, Michigan, United States of America; 7 Department of Computer Science and Engineering, Michigan State University, East Lansing, Michigan, United States of America; Deutsches Krebsforschungszentrum, Germany

## Abstract

**Background:**

Network analysis has been performed on large-scale medical data, capturing the global topology of drugs, targets, and disease relationships. A smaller-scale network is amenable to a more detailed and focused analysis of the individual members and their interactions in a network, which can complement the global topological descriptions of a network system. Analysis of these smaller networks can help address questions, i.e., what governs the pairing of the different cancers and drugs, is it driven by molecular findings or other factors, such as death statistics.

**Methodology/Principal Findings:**

We defined global and local lethality values representing death rates relative to other cancers vs. within a cancer. We generated two cancer networks, one of cancer types that share Food and Drug Administration (FDA) approved drugs (FDA cancer network), and another of cancer types that share clinical trials of FDA approved drugs (clinical trial cancer network). Breast cancer is the only cancer type with significant weighted degree values in both cancer networks. Lung cancer is significantly connected in the FDA cancer network, whereas ovarian cancer and lymphoma are significantly connected in the clinical trial cancer network. Correlation and linear regression analyses showed that global lethality impacts the drug approval and trial numbers, whereas, local lethality impacts the amount of drug sharing in trials and approvals. However, this effect does not apply to pancreatic, liver, and esophagus cancers as the sharing of drugs for these cancers is very low. We also collected mutation target information to generate cancer type associations which were compared with the cancer type associations derived from the drug target information. The analysis showed a weak overlap between the mutation and drug target based networks.

**Conclusions/Significance:**

The clinical and FDA cancer networks are differentially connected, with only breast cancer significantly connected in both networks. The networks of cancer-drug associations are moderately affected by the death statistics. A strong overlap does not exist between the cancer-drug associations and the molecular information. Overall, this analysis provides a systems level view of cancer drugs and suggests that death statistics (i.e. global vs. local lethality) have a differential impact on the number of approvals, trials and drug sharing.

## Introduction

Cancer is a complex disease, with many subtypes, affecting various tissues in diverse ways, thus giving rise to an abundance of chemotherapies. Taken together, cancers are the second leading cause of death in the United States [1]. The common features of cancer include uncontrolled cell growth, reduction in apoptosis, and loss of cell cycle regulation, while other features are more tissue specific and thus differentiate them and their chemotherapies.

In a global network level analysis of different diseases, where the vertices represented diseases and the edges represented connections between diseases that share common genetic background, most diseases were less connected, while a limited number of diseases, mostly cancers, were highly connected hubs [2]. Similarly, a network analysis of drugs, where the vertices represented drugs and the edges represented connections between drugs that share common protein targets, showed that drugs of similar types clustered together, and most proteins were targeted by a few drugs, whereas only a few proteins were targeted by many drugs [3], [4]. Cancers have fewer drugs that are used to treat them as compared with the other diseases, and the targets for the cancer drugs are at a shorter distance from the genes that are mutated in the cancers [3]. Quantitative analysis of the drug targets showed that proteins with at least 3 protein-protein interactions are more likely to be targeted by drugs [5]. A recent network study characterized the global map of many diseases, including cancers, and their associations with drugs, where the vertices represented diseases and the edges represented connections between diseases that share common drugs [6]. This study was also concerned with the global description of the network, and found that only a few diseases are highly connected by drugs, while most diseases are less connected; and most diseases, even those unrelated to each other, are connected by a few links [6]. These studies constitute the global topological analysis aspect of the emerging areas of network medicine [7] and network pharmacology [8]. However, these studies do not focus on the specific relationships between diseases and drugs, to address questions, such as, how might these relationships arise, or what factors may affect these relationships.

The field of medical sciences includes both basic molecular and clinical research, the latter involves clinical trials. Clinical trials apply biomedical protocols to humans that aim to intervene or observe a disease, e.g., testing drugs on cancers (http://clinicaltrials.gov). Clinical trials provide preliminary evidence of the efficacy, risks and optimum usage of the drugs. Phase 1 and 2 clinical trials are performed on small groups of individuals to evaluate their safety and efficiency. Phase 3 clinical trials are performed on a large group of individuals, to evaluate their efficiency, side effects and how they compare with approved drugs. Phase 4 clinical trials are performed after the drug has been approved for use, to obtain additional information. The United States Food and Drug Administration (FDA) regulates the approval and labeling of the drugs with regard to their safety, efficacy, and security to humans (http://www.fda.gov). In addition to the clinical drug trial and FDA approval data, death statistics, such as the estimated cases and estimated deaths over the years are available for the different cancer types [9]. Cancer is a large class of disease with various types, each with its own specific approvals, trials, death statistics, and molecular information, i.e., mutation targets. These diverse data provide opportunities to perform an integrative, systems level analysis of the cancers to reveal potential relationships between the various types of cancer and the drugs used to treat them and possible trends or factors that influence these relationships.

Global network analyses have been previously applied to describe the overall topology of disease and drug relationships, i. e, very few diseases and drugs are highly connected, while most members of these networks are less connected [2], [3], [4], [6].Smaller network systems, such as in this study, are amendable to a more focused analysis of individual members of the network, whereas larger networks are not, and hence are more amendable to statistical topological analyses, such as degree distribution analysis [10]. We propose that a drug approved or used in clinical trials for treating several cancers may hint to a relationship between those cancers. Similarly, a mutation involved in or a drug target used in treating different cancers may suggest a relationship between these cancers. System level analysis of these relationships could reveal potential factors involved in the development of these complex relationships that are not readily apparent from the data itself. In contrast to the previous medical network analyses, the analysis of smaller networks of cancer-drug and cancer-target associations permits a more detailed evaluation of the specific relationships between individual cancers. Through correlation and linear regression analyses of the number of approvals and trials, and weighted degree values, with the cancer lethality values, we assessed whether the death statistics impact the formation of associations between the cancers and drugs. Our analyses suggest that global lethality has an affect on the number of FDA approved and clinical trial cancer drugs. Comparative analysis of the cancer networks based on the FDA approved drugs and clinical trial drugs showed that some cancers are significantly and highly connected in the clinical trial cancer network but not in the FDA cancer network, and vice versa. Correlation and linear regression analyses suggest that local and global lethality differentially impact the sharing of FDA approved cancer drugs and the sharing of clinical trial drugs. Further, a comparison of the mutation target-based with the FDA drug target-based cancer networks suggests that the molecular information about a cancer does not strongly influence the cancer drug approvals.

## Results and Discussion

### FDA cancer drug approvals and clinical cancer drug trials

**Figure 1:**
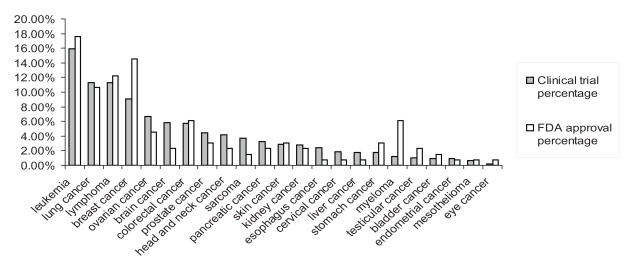
Cancer drug approval and clinical trial percentages. FDA cancer drug approval and clinical drug trial percentages for 23 cancers.

We collected the drugs approved through 2009 by the FDA for 23 cancer types and the clinical trials completed by 2009 for these same drugs (Dataset S1-S3). We compared these 81 drugs for the 23 cancer types, and checked which drugs had i) completed Phase 1 and 2 trials but were not listed under Phase 3 clinical trials and thus were not FDA approved, ii) completed Phase 3 clinical trial but were not FDA approved, iii) was FDA approved and in Phase 3 clinical trial (Table 1), and iv) was FDA approved and was not in clinical trials. There are several drugs for which Phase 3 clinical trial was completed but were not FDA approved (item ii). For example, cisplatin was approved for only testicular and bladder cancers, and has undergone and completed Phase 3 clinical trials for many types of cancer but has yet to be listed as approved by the FDA for those cancers (Table 1). The clinical trial data is incomplete (see Materials and Methods section for details). For example, there are some drugs which were FDA approved but not listed under any past clinical trials, completed or otherwise, which suggests that the analysis of the clinical trials will not be comprehensive. Leukemia, breast cancer, lung cancer, and lymphoma have the highest number of drug approvals and the highest number of clinical trials (Table 2, Figure S1). The percentage of clinical trials or FDA approvals for the different cancers were calculated as the number of clinical drug trials or FDA drug approvals for a specific cancer type, divided by the total number of clinical drug trials or FDA drug approvals for the 23 cancers analyzed in this study. The clinical trial and FDA approval percentages are similar for many of the cancers in this study (Figure 1). There are a few notable exceptions, namely breast cancer and myeloma, which have much higher percentages of FDA approvals than of clinical trials.

**Table 1:** Clinical trial and FDA approvals of drugs for different cancer types.

Cancer type	Phase 3 trial only	Phase 1,2 only	Phase 3 and FDA	FDA only
bladder cancer	doxorubicin,gemcitabine,paclitaxel	carboplatin,ifosfamide,bortezomib,trastuzumab,ixabepilone	cisplatin	-
brain cancer	procarbazine,cisplatin,ifosfamide,carboplatin,thalidomide,etoposide	cladribine,irinotecan,bortezomib,gefitinib,lenalidomide,busulfan,erlotinib,oxaliplatin,imatinib,temsirolimus,ixabepilone,topotecan,methotrexate,lapatinib,capecitabine,sorafenib	carmustine,temozolomide,cyclophosphamide	-
breast cancer	carboplatin,carmustine,cisplatin,doxorubicin,zoledronate,vinorelbine	decitabine,busulfan,etoposide,fludarabine,leucovorin,temozolomide,gefitinib,oxaliplatin,pemetrexed,dasatinib,irinotecan,bortezomib,erlotinib,imatinib,vorinostat,alemtuzumab	anastrozole,capecitabine,docetaxel,fulvestrant,gemcitabine,lapatinib,paclitaxel,trastuzumab,cyclophosphamide	epirubicin,pamidronate,toremifene,fluorouracil
cervical cancer	cisplatin	gemcitabine,capecitabine,paclitaxel,fluorouracil,oxaliplatin,arsenic trioxide,erlotinib,docetaxel,gefitinib	topotecan	-
colorectal cancer	cisplatin,doxorubicin	gefitinib,erlotinib,gemcitabine,trastuzumab,carmustine,ixabepilone,imatinib	bevacizumab,capecitabine,cetuximab,irinotecan,oxaliplatin,fluorouracil,leucovorin	panitumumab
endometrial cancer	cisplatin,doxorubicin,paclitaxel	capecitabine,topotecan,pemetrexed,oxaliplatin,raloxifene,thalidomide	-	methotrexate
esophagus cancer	cisplatin,fluorouracil	oxaliplatin,capecitabine,carboplatin,paclitaxel,irinotecan,topotecan,decitabine,vinorelbine,doxorubicin,docetaxel,erlotinib,arsenic trioxide,leucovorin,ixabepilone	-	porfimer
eye cancer	-	busulfan,carboplatin,topotecan	-	cyclophosphamide
head and neck cancer	cisplatin,fluorouracil,paclitaxel	irinotecan,oxaliplatin,bevacizumab,erlotinib,azacitidine,capecitabine,carboplatin,temozolomide,ixabepilone,doxorubicin,topotecan,carmustine,cyclophosphamide,etoposide,porfimer,thalidomide,gefitinib,bortezomib,sorafenib,gemcitabine	cetuximab,docetaxel	-
kidney cancer	carboplatin,cyclophosphamide,doxorubicin,etoposide	paclitaxel,irinotecan,oxaliplatin,temozolomide,busulfan,fludarabine,bevacizumab,cetuximab,erlotinib,fluorouracil,pentostatin,topotecan,arsenic trioxide,gefitinib,methotrexate,capecitabine,gemcitabine,lenalidomide,imatinib,denileukin diftitox,thalidomide	-	sunitinib
leukemia	cytarabine,etoposide,leucovorin,doxorubicin,ifosfamide	topotecan,bexarotene,carboplatin,bortezomib,temozolomide,rituximab,bevacizumab,sorafenib,thalidomide,denileukin diftitox,docetaxel,ixabepilone,temsirolimus	alemtuzumab,busulfan,daunorubicin,fludarabine,idarubicin,imatinib,mitoxantrone,methotrexate,cyclophosphamide	mechlorethamine,nilotinib,teniposide,bendamustine
liver cancer	cisplatin,fluorouracil,doxorubicin	irinotecan,oxaliplatin,temozolomide,erlotinib,gemcitabine,pemetrexed,capecitabine,carboplatin,topotecan,thalidomide,docetaxel,epirubicin	-	-
lung cancer	cisplatin,carboplatin	sunitinib,busulfan,cyclophosphamide,fludarabine,cetuximab,decitabine,imatinib,irinotecan,doxorubicin,bortezomib,ifosfamide,sorafenib,fluorouracil,azacitidine,trastuzumab,temozolomide,thalidomide,temsirolimus	docetaxel,erlotinib,etoposide,gefitinib,gemcitabine,paclitaxel,pemetrexed	mechlorethamine,nofetumomab,porfimer,methotrexate
lymphoma	etoposide,doxorubicin,ifosfamide,leucovorin,cisplatin,idarubicin,mitoxantrone,daunorubicin	topotecan,paclitaxel,irinotecan,oxaliplatin,busulfan,imatinib,temozolomide,fludarabine,cladribine,decitabine,alemtuzumab,arsenic trioxide,altretamine,gemcitabine,carboplatin,azacitidine,pentostatin,thalidomide,ixabepilone,temsirolimus,tretinoin,bevacizumab,sorafenib	carmustine,cytarabine,rituximab,methotrexate,cyclophosphamide	bexarotene,methoxsalen,procarbazine,vorinostat,bendamustine
mesothelioma	cisplatin	decitabine,doxorubicin,gemcitabine,epirubicin,gefitinib,bevacizumab	pemetrexed	-
myeloma	-	arsenic trioxide,fludarabine,etoposide,cisplatin,clofarabine	bortezomib,thalidomide,cyclophosphamide	carmustine,doxorubicin,lenalidomide,zoledronate
ovarian cancer	epirubicin,mitoxantrone	vinorelbine,temozolomide,docetaxel,ixabepilone,cisplatin,capecitabine,etoposide,ifosfamide,gemcitabine,bortezomib,lapatinib,erlotinib,imatinib,gefitinib,anastrozole,letrozole,pemetrexed,oxaliplatin,alemtuzumab,leucovorin,methotrexate,irinotecan,sorafenib,toremifene,bevacizumab,cetuximab,vorinostat	carboplatin,paclitaxel,cyclophosphamide	Altretamine
pancreatic cancer	oxaliplatin	lapatinib,irinotecan,bevacizumab,cetuximab,cisplatin,pemetrexed,imatinib,trastuzumab,capecitabine,leucovorin,paclitaxel,docetaxel,ixabepilone,bortezomib,arsenic trioxide,temsirolimus,cyclophosphamide	erlotinib,gemcitabine,fluorouracil	-
prostate cancer	mitoxantrone,zoledronate,toremifene	doxorubicin,paclitaxel,carboplatin,epirubicin,temsirolimus,ixabepilone,pemetrexed,oxaliplatin,sunitinib,azacitidine,imatinib,trastuzumab,arsenic trioxide,bevacizumab,thalidomide,lapatinib	leuprolide,degarelix	-
sarcoma	cisplatin,doxorubicin,etoposide,ifosfamide,daunorubicin,cyclophosphamide,topotecan	irinotecan,oxaliplatin,temozolomide,busulfan,erlotinib,carboplatin,altretamine,leucovorin,paclitaxel,thalidomide,gemcitabine,trastuzumab,ixabepilone,bevacizumab,cytarabine	methotrexate	-
skin cancer	cisplatin	lenalidomide,decitabine,irinotecan,oxaliplatin,busulfan,cyclophosphamide,etoposide,fludarabine,docetaxel,leucovorin,sorafenib,temozolomide,thalidomide,denileukin diftitox,tretinoin,carmustine,temsirolimus,bortezomib,ixabepilone	-	daunorubicin,doxorubicin,imiquimod
stomach cancer	-	irinotecan,cisplatin,gemcitabine,vinorelbine,doxorubicin,paclitaxel,leucovorin,oxaliplatin,ixabepilone,erlotinib,capecitabine	-	imatinib,sunitinib
testicular cancer	carboplatin,cyclophosphamide,paclitaxel	busulfan,fludarabine,temozolomide,topotecan,ixabepilone,alemtuzumab,arsenic trioxide,imatinib	etoposide,ifosfamide,cisplatin	-

**Table 2:** FDA approvals, clinical trial, weighted degree values and death statistics of cancers in this study.

Cancer type	FDA drug approval number	FDA specific drug approval number	FDA specific drug percentage	FDA original drug approval number	Clinical drug trial number	FDA cancer network weighted degree value	FDA weighted degree p-value	Clinical trial cancer network weighted degree value	Clinical trial weighted degree p-value	Global lethality ratio	Local lethality ratio
lung cancer	14	3	21.4%	1	121	1.32	0.029	7.58	0.066	0.286	0.753
colorectal cancer	8	4	50.0%	3	61	0.46	0.840	6.15	0.899	0.088	0.336
breast cancer	19	10	52.6%	2	97	1.17	0.003	6.81	0.037	0.072	0.222
pancreatic cancer	3	0	0.0%	1	35	0.5	0.898	6.96	0.576	0.061	0.910
prostate cancer	4	3	75.0%	0	48	0.41	0.937	4.32	0.998	0.051	0.154
leukemia	23	16	69.6%	0	170	0.65	0.157	5.16	0.656	0.038	0.490
lymphoma	16	9	56.3%	0	121	0.83	0.195	6.41	0.013	0.036	0.276
liver cancer	1	0	0.0%	0	19	0.33	0.899	6.57	0.714	0.033	0.862
endometrial cancer	1	0	0.0%	0	10	1.06	0.653	4.43	0.996	0.028	0.186
ovarian cancer	6	2	33.3%	2	71	1.16	0.488	7.42	0.018	0.027	0.717
esophagus cancer	1	0	0.0%	1	26	0.07	0.970	6.8	0.605	0.025	0.867
bladder cancer	2	1	50.0%	0	10	0.25	0.967	4.34	0.997	0.025	0.205
brain cancer	3	1	33.3%	1	62	0.89	0.748	6.57	0.606	0.023	0.599
kidney cancer	3	1	33.3%	1	30	0.5	0.899	7.22	0.221	0.023	0.239
skin cancer	4	1	25.0%	2	31	0.48	0.885	5.84	0.924	0.020	0.165
myeloma	8	3	37.5%	1	13	0.86	0.563	2.81	1.000	0.019	0.537
stomach cancer	4	0	0.0%	1	19	1.13	0.609	6.32	0.759	0.019	0.506
cervical cancer	1	0	0.0%	0	20	0.24	0.939	5.48	0.932	0.007	0.350
testicular cancer	3	1	33.3%	2	11	0.31	0.966	5.31	0.984	0.001	0.047
eye cancer	1	0	0.0%	0	2	0.78	0.691	1.58	1.000	0.000	0.100
head and neck cancer	3	0	0.0%	0	45	1.35	0.558	8.03	0.056	-	-
mesothelioma	1	0	0.0%	0	40	0.07	0.984	3.23	1.000	-	-
sarcoma	2	0	0.0%	1	7	1.21	0.636	7.39	0.259	-	-

### Global and local lethality values for cancer types

Death and survival ratios have been predominantly used to describe the values of global and local significance of cancer deaths [9]. It is confusing to use these values since one uses death and the other uses survival numbers to describe global and local death statistics of a specific cancer. Therefore, we defined two different death-based statistics, a global and a local lethality rate by using the estimated death and new case numbers of each cancer (Table 2 and S1). The percentage of global lethality is calculated as the ratio of estimated number of deaths for a cancer to the estimated number of deaths for all cancers. The percentage of local lethality is calculated as the ratio of estimated number of deaths to the estimated number of cases for a particular cancer. The global lethality provides a perspective of a particular cancer with respect to the other cancers, whereas, the local lethality is specific to each cancer type. A cancer with a high local lethality suggests that it has a high number of deaths within its own incidences, while its global lethality may or may not be high. For example, pancreatic cancer is a locally lethal but not globally lethal cancer; it has a local lethality value of 0.91 but a global lethality value of 0.06 (Table 2). This is because most of the pancreatic cancer patients have low survival rates, but comparatively there are fewer cases of pancreatic cancer.

### Effect of lethality on FDA approvals and clinical trials

We hypothesize that there are factors, such as the lethality values of a cancer, that may influence the number of clinical trials and, in turn, FDA approvals. To quantitatively evaluate whether lethality values are related to the number of FDA drug approvals and clinical drug trials, Spearman correlation coefficients were calculated between the global/local lethality measures and the trial/approval numbers. The correlation analyses suggest that global lethality is correlated, whereas local lethality is not correlated, to both the clinical trial and FDA approval numbers (Table 3). To further evaluate the impact of lethality values on the FDA drug approvals and clinical drug trials, we performed a linear regression analysis. Linear fit of the clinical trial numbers with global lethality suggests a slight but albeit significant relationship (r^2^ = 0.25, p = 0.03, Figure S2). This suggests the higher clinical drug trial numbers could be explained, in part, by the higher global lethality rates. Next, we considered whether the relationships found by correlation and linear regression analyses are affected by lung cancer, the most globally lethal cancer, and pancreatic, esophagus, and liver cancers, the most locally lethal cancers (see Table 2 and the Materials and Methods section). We re-calculated the correlations by removing the globally or locally lethal cancers. No significant change in the correlations resulted upon removing lung cancer. However, a linear fit of the FDA approval numbers with global lethality suggests a slight relationship which is significant, when lung cancer is excluded (r^2^ = 0.20, p = 0.05, Figure S2). The significance of the correlation and the linear fit between local lethality with FDA approval and clinical trial numbers increased upon removing the most locally lethal cancers, pancreatic, liver and esophagus cancers (Figure S2, Table 3). Local lethality has a significant correlation with clinical trial drug numbers for the cancers other than the most locally lethal ones. This suggests the number of FDA approvals and clinical trials are much lower for pancreatic, liver and esophagus cancers as compared to other cancers despite their very high local lethality (Text S1). Although, the linear fit p-values of local lethality with FDA approval numbers and clinical trial numbers decreased, when pancreatic, liver and esophagus cancers are excluded, they are not very significant (Figure S2). We also analyzed whether the FDA approval numbers from previous years correlated with the lethality values. The correlation of global lethality with the FDA approval numbers has mostly been present in previous years (Table S2). The correlation and linear regression analyses suggest that global lethality has an impact on the drug trial and approval numbers, for the cancers in this study.

**Table 3:** Correlation values of weighted degree, approval number values and of FDA specific drug percentage with global and local lethality values.

	Global lethality		Local lethality		
	All cancer types	All cancer types except globally lethal cancers (lung cancer)	All cancer types	All cancer types except locally lethal cancers (pancreatic, esophagus and liver cancers)	
**FDA approval number**	0.50	0.44	0.05	0.42	Spearman statistic
	0.03	0.06	0.85	0.09	Spearman p-value
**Clinical trial number**	0.67	0.63	0.34	0.53	Spearman statistic
	0.00	0.00	0.15	0.03	Spearman p-value
**FDA cancer network weighted degree**	0.25	0.12	0.14	0.53	Spearman statistic
	0.29	0.62	0.57	0.03	Spearman p-value
**Clinical trial cancer network weighted degree**	0.42	0.33	0.61	0.55	Spearman statistic
	0.06	0.17	0.00	0.03	Spearman p-value
**FDA specific drug percentage**	0.35	0.44	−0.32	−0.05	Spearman statistic
	0.13	0.06	0.17	0.85	Spearman p-value

### Weighted cancer networks

The global relationships between drugs and diseases have been analyzed topologically in large-scale networks of drugs and diseases [2], [3], [4], [6]. Complex relationships between the types of cancer and drugs constitute a smaller network structure. Unlike the larger networks, a smaller network system, as in this study, are amendable to a more focused analysis of individual members of the network rather than statistical topology-based parameters [10]. We applied this more focused analysis, where individual members and interactions in the networks were studied rather than their global structure, to elucidate the drug therapy based relationships between various cancers and the factors that may influence these relationships.

**Figure 2:**
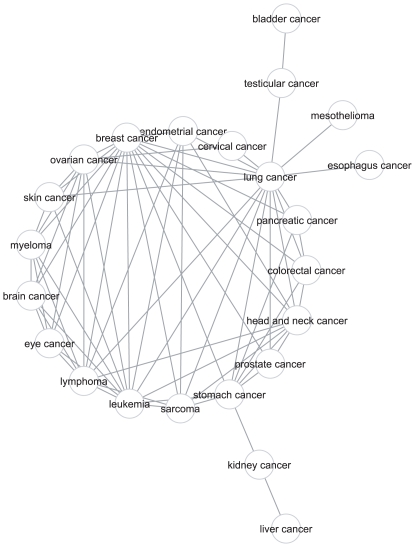
FDA cancer network. Vertices represent cancers whereas edges represent the drug approval-based interaction between them. The network includes only the cancers which have at least one interaction with other cancers.

**Figure 3:**
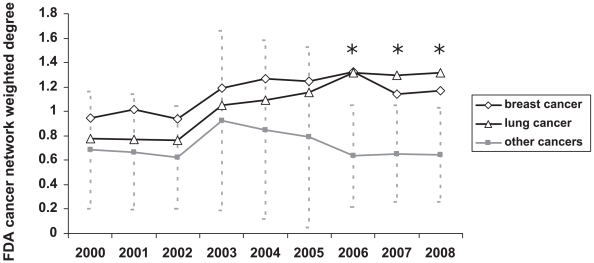
Weighted degree values breast and lung cancers in the previous years. Weighted degree values of breast cancer, lung cancer, and the remaining cancers in the FDA cancer networks from 2000 to 2008. Average and the standard deviation of the weighted degree values are shown. Wilcoxon test was performed for greater values of lung and breast cancer than the other cancers. The networks with p-values lower than 0.05 are indicated by asterisk (*).

The collection of cancer-drug pairs make up a bipartite network, which we transformed into a unipartite weighted network consisting of only cancers. To construct a weighted network of cancers, an edge between any two cancers was assigned, if there is at least one drug which was approved by FDA to treat both types of cancers (Figure 2, Dataset S1, Table S3). The weight of an edge was defined by the Jaccard index, which is the fraction of drugs which were approved for both cancers over all the drugs which were approved for each of the two cancers, separately (see Materials and Methods). Weighted degree values were not significantly correlated with the number of FDA approvals (Pearson correlation coefficient of 0.34, p = 0.11), suggesting that the number of drugs approved for a cancer does not implicate the number of drugs shared with other cancers. We, further, assessed the significance of the weighted degree values by a permutation test, while keeping the number of drugs per cancer type constant, and found the degree of drug sharing is not significant for most of the cancers (Table 2), except for lung and breast cancer These two cancers have significant weighted degree values in the FDA cancer network. Lung cancer shares FDA drugs with many other cancers (Figure 2, Table S3). Leukemia, the cancer type with the highest number of FDA approvals, does not have a significant weighted degree value in the FDA cancer network (Table 2). This is because leukemia does not share many of its FDA approved drugs with other cancers. Indeed, as discussed later, leukemia has many specific drugs (see section “Drugs specific to particular cancer types”). We also analyzed the FDA cancer network over time, by including the cancer drug approvals for the different years (Figure S3-S4, Text S1). Based on the average weight values of the networks, there is no major change over the years. Weighted degree values for most of the cancers also are not significant in the previous years' networks. However the breast cancer weighted degree value has been significant since 2000 and the lung cancer degree value has become significant recently (Table S4). Weighted degree values of lung and breast cancer have been increasing and significantly higher than the other cancers since 2006 (Figure 3). In recent years, FDA approved drugs for these cancers (the 1^st^ and 3^rd^ most globally lethal) have a high overlap with other cancers.

A weighted clinical trial-based cancer network was also constructed (herein denoted as clinical trial cancer network), where two cancers were connected if there is at least one FDA approved drug (approved for at least a cancer) in the clinical trial data for both cancers (Dataset S3, Table S3). The clinical trial cancer network is almost a complete network, because of the large number of drugs that were used in clinical trials for the different cancers, thereby connecting many of the cancers, albeit not all, to each other (Figure S5). The significance of the weighted degree values was evaluated by a permutation test, with the number of drug trials kept constant. Breast cancer, ovarian cancer, and lymphoma have significant weighted degree values in the clinical trial cancer network (Table 2). Also, the weighted degree values of lung cancer and head and neck cancer are close to being significant. This indicates that these cancers shares clinical trial drugs, significantly, with other cancers. In addition, we calculated the difference in the edge weights between the FDA and clinical trial cancer networks for each cancer pair, and identified that most pairs are strongly connected in the clinical trial but not in the FDA cancer network (Table S3). For example, stomach and esophagus cancers are strongly connected in the clinical trial cancer network (Table 4). There are many drugs used in clinical trials for both types of cancers, i.e., capecitabine, cisplatin, doxorubicin, erlotinib, fluorouracil, irinotecan, ixabepilone, leucovorin, oxaliplatin, paclitaxel, and vinorelbine, and thus strongly connecting these two cancers. However, they are not connected in the FDA cancer network, i.e. no drug is approved by the FDA for both stomach and esophagus cancers; porfimer was approved for esophagus cancer while docetaxel, fluorouracil, imatinib, and sunitinib were approved for stomach cancer. There are a few pairs of cancers which are more highly connected in the FDA cancer network than in the clinical trial cancer network (Table 4 and S3). For example, sarcoma and endometrial cancer pair has a weight of 0.5; they share methotrexate which is the only drug approved for endometrial cancer and one of the two drugs approved for sarcoma. On the other hand, there are many drugs in the clinical trial data for each of these two cancers which are not shared between them, such as altretamine, capecitabine, etoposide, etc (Dataset S3). Weighted networks of cancers based on FDA approvals and clinical trials show different characteristics. Breast cancer is the only cancer with a significant degree value in both the FDA and the clinical trial cancer networks. While lung cancer is more significantly connected only in the FDA cancer network, ovarian cancer and lymphoma are more significantly connected in the clinical trial cancer network (Table 2). This suggests that ovarian cancer and lymphoma have a high overlap of drugs in clinical trials but not in FDA approvals.

**Table 4:** Cancer pairs with a weight difference of at least 0.5 or lower than 0.

Cancer type 1	Cancer type 2	Clinical trial cancer network weight	FDA cancer network weight	Difference
stomach cancer	esophagus cancer	0.71	0.00	0.71
head and neck cancer	kidney cancer	0.56	0.00	0.56
kidney cancer	lung cancer	0.54	0.00	0.54
ovarian cancer	head and neck cancer	0.54	0.00	0.54
leukemia	lymphoma	0.68	0.15	0.53
ovarian cancer	breast cancer	0.61	0.09	0.53
cervical cancer	esophagus cancer	0.50	0.00	0.50
head and neck cancer	brain cancer	0.50	0.00	0.50
head and neck cancer	liver cancer	0.50	0.00	0.50
stomach cancer	cervical cancer	0.50	0.00	0.50
brain cancer	myeloma	0.17	0.22	−0.05
ovarian cancer	myeloma	0.10	0.17	−0.06
head and neck cancer	endometrial cancer	0.25	0.33	−0.08
ovarian cancer	eye cancer	0.06	0.17	−0.11
eye cancer	myeloma	0.00	0.13	−0.13
brain cancer	eye cancer	0.12	0.33	−0.21
sarcoma	endometrial cancer	0.22	0.50	−0.28

### Effect of lethality on the cancer networks

Given that the lethality of a cancer impacts the number of drug trials and approvals, it raises the question of whether it could also influence the FDA and clinical trial cancer networks and if there could be differences in their influence on these two networks. We analyzed the correlation and the linear fit between the weighted degree values of the FDA/clinical trial cancer networks and the global/local lethality values. The weighted degree values for the clinical trial cancer network are correlated with local lethality (Table 3). Linear regression between the weighted degree values and the lethality values shows a partial but significant relationship between local lethality and clinical trial network weighted degree (r^2^ = 0.26, p = 0.02, Figure S6). This suggests that sharing of drugs in clinical trials is impacted positively by local lethality values.

**Figure 4:**
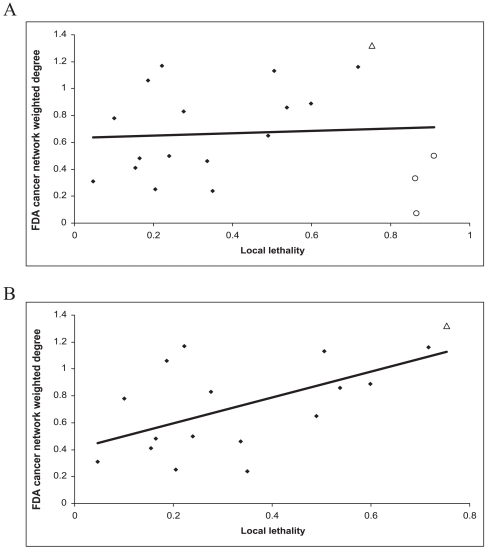
FDA cancer network weighted degree vs. local lethality ratio. FDA cancer network weighted degree values are plotted against local lethality ratio for (**A**) 23 cancers (r^2^ = 0.01, p = 0.78), (**B**) the cancers except pancreatic, liver and esophagus cancers (r^2^ = 0.35, p = 0.01). Lung cancer is shown as an open triangle and pancreatic, liver, esophagus cancers are shown as open circles.

The weighted degree values of the FDA cancer network are not significantly correlated with the global and local lethality values (Table 3). Next, we analyzed the effect of the most globally lethal (lung cancer) and the most locally lethal cancers (pancreatic, esophagus and liver cancers) on these correlations and linear fits. Weighted degree values of the FDA cancer network are significantly correlated with local lethality after removing pancreatic, liver, and esophagus cancers (Table 3, Figure 4A–4B). Linear fit analysis suggests that the weighted degree of a cancer in the FDA cancer network tend to be high if its local lethality value is high. However, the most locally lethal cancers (pancreatic, esophagus and liver cancers) are excluded from this effect since they have lower than expected weighted degree values, as compared to the other cancers (Text S1). We also analyzed if the FDA cancer networks from previous years correlated with the lethality values. Global lethality and local lethality do not have a significant correlation in the older FDA cancer networks. However, more recently (2007) the cancer network has become correlated with local lethality, with the exclusion of pancreatic, liver, and esophagus cancers (Table S2).

Analysis of the weighted degree values of the cancer networks provides information on the level of drug sharing between cancers. We showed that local lethality has an effect on the clinical cancer drug trial sharing as well as FDA approved drug sharing, the latter appears to be a recent trend. However, the most locally lethal cancers, pancreatic, liver, and esophagus cancers, are biased towards having lower levels of sharing of FDA approved drug. For the most local lethality cancers, although sharing of drugs in clinical trials correlates positively with local lethality values, the sharing of the approved drugs does not correlate with local lethality values.

### Specific and originally approved drugs to particular cancer types

**Figure 5:**
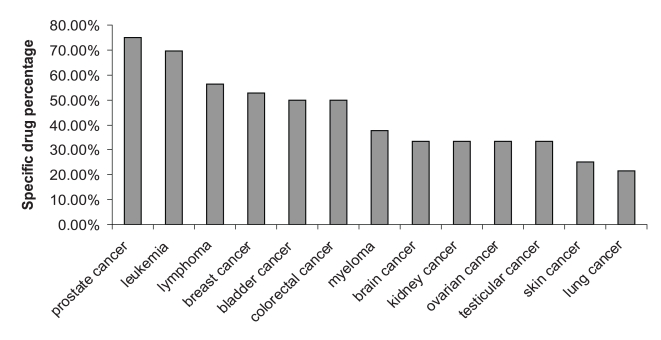
FDA specific drug percentages. FDA and clinical trial specific drug percentages for the cancers except cervical, endometrial, esophagus, liver, pancreatic, eye, sarcoma, mesothelioma, and stomach cancers, which do not have specific drugs.

Network analysis captured the overlap in cancer drug use, however, only 26 of the total 81 cancer drugs were approved for more than one cancer type. Therefore we analyzed the distribution of the remaining 55 drugs which were approved specifically for only one type of cancer. A drug which was approved by the FDA solely for a single cancer is denoted as a “specific” FDA drug. We calculated the specific drug percentage for a cancer as the ratio of the number of specific drugs to the total number of drugs approved by the FDA. Prostate cancer, leukemia, breast cancer, and lymphoma have the highest specific drug percentage approved by the FDA (Figure 5). The most locally lethal cancers, pancreatic, liver, and esophagus cancers, have no specific drugs (Table 2). Globally lethal cancer, i.e., lung cancer, has a low percentage of FDA specific drugs (Table 2, Figure 5). The number of specific drugs in clinical trials is very low, therefore it was not analyzed further (Table S5). We also analyzed the possible effect of lethality on the percentage of FDA specific drug approvals and showed that there is no significant effect based on correlation and linear regression analyses (Table 3 and Figure S7).

There is also a notable difference among the non-specific (shared) drugs, such that some of the drugs were first approved for a cancer type and then approved for other cancer types, while other drugs might be approved for more than one type of cancer at the same time. We defined whether a drug was “originally approved” by the FDA for a specific cancer type and then approved for other cancers after at least a year. Colorectal cancer has the highest number of “originally approved” FDA drugs (Table 2). There is only one originally approved FDA drug, erlotinib for lung cancer (Table 2, Dataset S1). Many more drugs were approved for other cancers that were subsequently approved for lung cancer (11 drugs) than were “originally approved” for lung cancer (only one).

### Comparison of clinical and molecular target based cancer networks

In addition to the death statistics, we asked whether molecular information impacted the cancer-drug associations. To compare the molecular target-based relationships to the clinical target-based relationships for the different cancer types, we constructed weighted molecular and clinical cancer networks (Figure S8) based on mutation targets and FDA approved drug targets (Dataset S4–S6, Table S6–S7), respectively. The edges between two cancers in the mutation target based network was assigned if there is at least one mutation target associated with both cancers and the edges between two cancers in the drug target based network was assigned if there is at least one drug target associated with both cancers. The weights of the edges were defined by the Jaccard index (Table S6–S7). To compare the mutation target-based and the drug target-based cancer networks, we included only the cancers that have both mutation and drug target data. We calculated the weighted degree values for the different cancers and evaluated the significance of the weighted degrees with permutation test, keeping the distribution of target numbers for each cancer constant (Table 5). The weighted degree values of the mutation and drug-target based cancer networks are not strongly correlated (Pearson correlation coefficient of 0.37, p = 0.11). Lung and breast cancers have significant and high weighted degree values in the drug target-based network but not in the mutation target-based network (Table 5). On the other hand, colorectal, ovarian and brain cancers have significant weighted degree values in the mutation target-based network but not in the drug-target based network (Table 5). Leukemia is the only cancer which has significant weighted degree values in both networks.

**Table 5:** Weighted degree values of drug target and mutation target based networks.

	Drug target based network weighted degree value	Drug target based network weighted degree p-value	Mutation target based network weighted degree value	Mutation target based network weighted degree p-value
leukemia	2.28	0.000	0.25	0.003
lung cancer	3.35	0.000	1.12	0.210
breast cancer	3.29	0.001	1.08	0.160
colorectal cancer	2.75	0.290	1.57	0.000
ovarian cancer	2.53	0.634	1.43	0.011
brain cancer	1.66	0.674	0.9	0.016
sarcoma	1.76	0.961	0.42	0.509
pancreatic cancer	0.62	1.000	0.82	0.706
endometrial cancer	0.66	0.996	0.78	0.732
eye cancer	1.79	0.309	0.17	0.880
stomach cancer	2.12	0.774	0.53	0.909
lymphoma	2.17	0.287	0.19	0.952
testicular cancer	1.83	0.817	0.33	0.980
skin cancer	2.22	0.808	0.35	0.981
bladder cancer	2.32	0.330	0.14	0.998
head and neck cancer	2.75	0.275	0.06	1.000
kidney cancer	1.38	0.996	0.13	1.000
liver cancer	1	0.999	0.26	1.000
myeloma	1.24	0.999	0.16	1.000
prostate cancer	0.88	1.000	0.19	1.000

The overlap between the two networks is very low (Figure S8) and for the overlapping edges, we calculated the difference in mutation and drug target weight values (Table 6 and S8). Colorectal-ovarian, ovarian-endometrial, and endometrial-colorectal cancer pairs have higher mutation target-based weights than drug target-based weight values (Table 6). These cancers are connected to each other in the mutation target-based network through the following mutations: PMS1, PMS2, MLH1, MSH2, and MSH6, which are proteins responsible for DNA mismatch repair. On the other hand, all three cancers share no drug targets. Since they share many mutation targets, this suggests that they could have similar molecular mechanisms, and thus raises the question if they should share drug targets. On the other hand, kidney and liver cancers, which do not share any mutation targets, have a high overlap of drug targets (Table S8). They share drug targets such as FLT4, PDGFRB, BRAF, etc. (Dataset S5). There could be mutation targets common to these cancers which may not have been identified or is absent in the current dataset. Alternatively, they could share molecular mechanisms without sharing mutation targets, i.e., similar pathways may be affected in both cancers despite different mutated genes.

**Table 6:** Mutation target- and drug target-based weight values of cancer pairs which have a positive difference between the drug and mutation target-based values.

Cancer type 1	Cancer type 2	Difference of mutation target from drug target based weight
colorectal cancer	endometrial cancer	0.26
colorectal cancer	ovarian cancer	0.24
endometrial cancer	ovarian cancer	0.15
brain cancer	colorectal cancer	0.14
ovarian cancer	pancreatic cancer	0.14
colorectal cancer	liver cancer	0.1
brain cancer	sarcoma	0.09
brain cancer	endometrial cancer	0.08
breast cancer	stomach cancer	0.08
endometrial cancer	stomach cancer	0.08
liver cancer	pancreatic cancer	0.08
pancreatic cancer	testicular cancer	0.08
brain cancer	lung cancer	0.07
colorectal cancer	pancreatic cancer	0.06
head and neck cancer	kidney cancer	0.06
liver cancer	ovarian cancer	0.06
brain cancer	prostate cancer	0.05
endometrial cancer	prostate cancer	0.05
eye cancer	lung cancer	0.05
lung cancer	stomach cancer	0.05
brain cancer	breast cancer	0.03
brain cancer	liver cancer	0.03
brain cancer	pancreatic cancer	0.03
brain cancer	stomach cancer	0.03
breast cancer	eye cancer	0.03
breast cancer	kidney cancer	0.03
colorectal cancer	sarcoma	0.03
pancreatic cancer	skin cancer	0.03
bladder cancer	sarcoma	0.02
brain cancer	kidney cancer	0.02
breast cancer	sarcoma	0.02
eye cancer	sarcoma	0.02
prostate cancer	sarcoma	0.01

We also evaluated the cancers that are associated with proteins that are both mutation and drug targets (Table 7). Only leukemia, lung, and breast cancers have mutation targets that are also drug targets. For example, ERBB2, a member of EGFR family, has long been known as a mutation target for breast cancer [11]. Lapatinib, letrozole, and trastuzumab are drugs that target ERBB2 in our data (Dataset S4) and all have been used in clinical drug trials for only breast cancer and approved by the FDA for only breast cancer (Dataset S1 and S3). Furthermore, ERBB1, a member of the EGFR family, is known as a mutation target for lung cancer [12]. There are several drugs which target ERBB1, such as cetuximab, erlotinib, gefitinib, lapatinib, panitumumab, and trastuzumab (Dataset S4), among which, only erlotinib and gefitinib are approved only for lung cancer (Dataset S1). The remaining drugs have not completed Phase 3 clinical trials. Cetuximab, and trastuzumab have completed Phase 1 and 2 trials, whereas clinical trials using lapatinib and panitumumab for lung cancer have not yet completed Phase 1 and 2 trials (Table 1). Overall, very few mutations have been approved as targets for cancer therapy.

**Table 7:** Cancers with at least one common mutation and drug target.

Cancer type	Common mutation and drug target name and Entrez Gene ID
lung cancer	ERBB1(1956)
breast cancer	ERBB2(2064)
leukemia	FCGR2B(2213), ABL1(25), PDGFRB(5159), KIT(3815), ABL2(27), LCK(3932), BCL2(596)

Comparison of mutation and drug-target based cancer networks indicate that the overlap is very low. Various cancers have strong associations in one but not in the other network. For instance, lung and breast cancers have significant drug-target based associations but not mutation-target based associations. Similarly there are pairs of cancers, such as the pair of colorectal and endometrial cancers, with relatively high weights in the mutation-target based network but not in the drug-target based network. This analysis suggests that the influence of molecular information on the cancer-associations is not strong, and there are very few proteins which are both mutation-targets and drug-targets.

### Conclusion

In this study, we present a systems level view of the cancer drugs. Comparing clinical trial and FDA approval based cancer networks, we showed that only breast cancer is significantly connected in both networks. Lung cancer is significantly connected in the FDA cancer network, whereas ovarian cancer and lymphoma are significantly connected in the clinical trial cancer network. This suggests that lung cancer has a high degree of sharing of FDA approved drugs with the other cancers. Indeed, it has the highest number of FDA approved drugs which are shared with other cancers. In contrast, ovarian cancer and lymphoma have a high degree of drug sharing in clinical trials but not in FDA approvals.

We also assessed whether death statistics and molecular information are related to the cancer-drug associations. We showed that the cancer-drug associations are differentially impacted by the type of lethality. Global lethality appears to have an affect on the number of FDA approved drugs and clinical drug trials, but not on the FDA approval and clinical trial-based drug sharing, as determined by the cancer network weighted degree values. On the other hand, local lethality has an affect on the FDA approval and clinical trial-based drug sharing, but not on the number of FDA approved drugs and clinical drug trials. The effect of local lethality on the sharing of FDA approved drugs is not present or captured by the most locally lethal cancers, pancreatic, liver and esophagus cancers. These cancers are biased towards having very low overlap of FDA approved drugs with other cancers. For example, there is only one drug approved for liver cancer, Sorafenib, which is shared with lung cancer; however there are 13 more FDA approved drugs for lung cancer, which are not approved for liver cancer, leading to the lower weight for liver cancer (Dataset S1, Table S3). Although sharing of drugs in clinical trials correlates positively with local lethality values, however, it does not translate to increase sharing of the approved drugs for the most locally lethal cancers. There could be a number of reasons for this; the drugs in clinical trial are not being approved for the most locally lethal cancers or they have not been approved yet. For example, liver cancer and lung cancer share 13 drugs out of total 32 drugs used in clinical trials for these cancers (Dataset S3). 5 of these 15 common/overlapping clinical trial drugs are approved for lung cancer by FDA but they are still in clinical trials for liver cancer. Therefore they have a higher connection weight in the clinical trial cancer network than the FDA cancer network (Table S3). These findings support network-based analysis and their ability to reveal relevant information distinct from the raw data. It is not surprising that clinical decisions may be impacted by death statistics. However, it is interesting that different types of death statistics (global lethality vs. local lethality) show different results. It should be kept in mind that this study does not capture all aspects of the clinical drug data. For example, this analysis does not account for the differential efficiencies of the various drugs used in treating a particular cancer, which could have an impact on why some cancers have few while others may have many more drugs that target it. The current analysis of the clinical trials is limited to those which have already been approved by the FDA for at least one cancer type and therefore do not include all cancer drugs currently in clinical trial.

Currently, most cancer drugs are designed to target the general mechanisms of cell division, which may not directly address the specific molecular mechanisms that drive the development of the type of cancer it aims to treat. We compared mutation and drug targets for various cancer types. We identified a number of differences and noted that some cancer types share mutation targets but not drug targets while others share drug targets but not mutation targets, thereby hinting at the possibility that new drug targets or mutation targets could be identified for these cancers. Nevertheless, there are many other factors to consider when evaluating the data. Although two cancer types may not have the same mutation targets, they may have the same genes that are differentially expressed, which could suggest the involvement of similar molecular mechanisms. Given that cancer treatment includes surgery, radiotherapy in addition to chemotherapy (http://www.cancer.gov/cancertopics/treatment/types-of-treatment) thus, this study provides a systems level analysis of the trends of one aspect of clinical cancer research, namely from the perspective of the drugs that are FDA approved or undergoing clinical trials.

In closing, we demonstrated a systems level view of the drugs that have been approved and how they have been shared between cancer types. Thus we envision that this study could be informative to medical researchers from both the basic and clinical sciences alike. The trends revealed in this study could be monitored in the following years for any changes and these analyses could be used to guide more in-depth analysis of potential targets that could be involved in future clinical cancer drug trials and approvals. For example, one could followed whether the FDA approved drug sharing continues to be significant for breast and lung cancers which appears to be recent trends, beginning in the 2000s, and whether the overlap between the molecular target based and the drug target based cancer networks increases.

## Materials and Methods

### Drug-cancer pairs

We obtained lists of cancer drugs from the National Cancer Institute Drug Information Summaries (http://www.cancer.gov/cancertopics/druginfo/alphalist), and the FDA Center for Drug Evaluation and Research (http://www.fda.gov/Drugs/default.htm). We used the indication information by 2009 from the drug labels from the Drugs@FDA database (http://www.accessdata.fda.gov/scripts/cder/drugsatfda/index.cfm?fuseaction=Search.Search_Drug_Name) to generate a list of drug-cancer associations (Dataset S1) that included 23 types of cancer. We renamed some cancers, for example, Kaposi's sarcoma is listed under skin cancer, glioma is listed under brain cancer, and different types of leukemia and lymphoma are listed more generally as leukemia and lymphoma, respectively. The time information tag of the FDA approved label files is also used. Drugs discontinued in the market were excluded. We obtained clinical trial information for all drug trials completed by 2009 from the Clinical Trials database (http://clinicaltrials.gov) (Dataset S2) and collected the clinical trials for the drugs and the cancer types that are in the list of FDA data (Dataset S3). We differentiated between Phase ½ and Phase 3 trials since the Phase ½ are initial trials on small groups of patients, whereas Phase 3 trials are performed on large groups of patients. We excluded Phase 4 trials since they are post-approval. We did not include neoplasms in our analysis. Names of drugs and cancers have been organized according to the FDA data. In addition, we only collected the trials which were listed as drug trials. These limitations could lead to loss of information, such that we have FDA approval information for some drugs without completed clinical trial information (Table 1). We observed that these limitations could affect the clinical trial information prior to 2000s, namely, there could be cases in which there is an approval of a drug earlier than the trial dates. Therefore, we did not perform a time analysis of the clinical trials.

### Cancer death and survival statistics

The cancer statistics for 2001–2008 of the estimated number of new cases and the estimated number of deaths for the different types of cancers were obtained from the American Cancer Society [9]. We defined two kinds of lethality values. Global lethality is defined as the ratio of deaths of a particular cancer over all cancers. Local lethality is defined as the ratio of deaths of a particular cancer over the cases of that particular cancer. For breast, ovarian, cervical cancers only the female population values were considered. Likewise, for prostate and testicular cancers only the male population values were used. For the other cancer types, both the male and female population values were included (Table S1). Lung cancer has the highest global lethality value, whereas pancreatic cancer has the highest local lethality value. To determine which other cancers are similar to lung and pancreatic cancers with respect to their global and local lethality values, we performed hierarchical clustering, based on Euclidean distance of lethality values with single linkage. Lung cancer clustered by itself, and pancreatic, liver and esophagus cancers clustered together (Figure S9). Therefore only lung cancer is considered globally lethal cancer, whereas pancreatic, liver and esophagus cancers are considered locally lethal cancers.

### Network construction

We constructed weighted clinical networks of cancer types, FDA cancer network and clinical trial cancer network, from the drug-cancer pairs (Dataset S1 and S3). In the clinical cancer networks an edge was defined between two cancer types when there is at least one drug which was approved or used in clinical trials for both types of cancer (Table S3). The weight of the edge was defined by the Jaccard index, which is the fraction of common drugs for both cancer types over all the drugs for each of the cancer types. For example, there is only one drug which was approved for both pancreatic and stomach cancers, fluorouracil, whereas there are 2 more drugs, erlotinib and gemcitabine, which were approved for pancreatic cancer but not for stomach cancer, and there are 3 more drugs, docetaxel, imatinib, and sunitinib, which were approved for stomach cancer but not for pancreatic cancer (Dataset S1). Therefore the weight of the edge between these two cancers is 1/(1+2+3) = 0.17 (Table S3). The resulting FDA drug approval-based cancer network (herein denoted as FDA cancer network) contains 23 types of cancer (vertices or nodes) with 70 interactions (edges). We defined the weighted degree value for a cancer as the sum of the weights of the edges for that cancer. For example, pancreatic cancer shares drugs with stomach, lung, colorectal and breast cancers, therefore its weighted degree is the sum of the weights of the edges with these cancers, which is 0.17+0.13+0.1+0.1 = 0.5 (Table 1 and S3). This parameter provides an account of the allocation of drugs for a particular cancer and its neighbors in the network. If more drugs, which are approved for other cancers, are approved for pancreatic cancer (regardless of whether the drug is shared with stomach, lung, colorectal and breast cancers or other cancers) in the future, its weighted degree value will increase. Its weighted degree value will decrease if more drugs are approved for stomach, lung, colorectal and breast cancers but not for pancreatic cancer.

Similarly, we also constructed molecular target and clinical target-based cancer networks (Table S6–S7), using mutation target data from the Cancer Gene Census database (http://www.sanger.ac.uk/genetics/CGP/Census/) [13] and FDA approved drug target data from the DrugBank database (http://www.drugbank.ca) (Dataset S4), respectively. Mutation target data from the Cancer Gene Census database used was updated in January 2009 and includes mutation targets which have been implicated in the cancer. This database was chosen because it is based on literature curation and thus captures information on the molecular mechanisms that clinical researchers should have information on. Cytoscape version 2.4 was used to visualize the networks [14].

### Statistical analysis

The significance of the weighted degree values in the cancer networks was analyzed by permutation tests. The distribution of the number of drugs or drug and mutation targets was kept constant while the cancer-drug or cancer-target associations were randomized, respectively. The p-value for the weighted degree of a cancer type is calculated as the fraction of the randomly generated networks with a weighted degree value for a particular cancer which is equal to or greater than the actual weighted degree value of that particular cancer (Table 2 and 5). Conventional cutoff of 0.05 was used as a significance threshold. No multiple test correction has been applied to the p-values. Therefore, given the number of statistical tests performed, some of the associations reported, particularly borderline significant, could be spurious. In the FDA cancer network, Wilcoxon test is used to determine if the weighted degree values of breast and lung cancer are higher than the rest of the cancers in the network.

Shapiro-Wilk test suggests that some of the datasets used in this study are not normally distributed (see Text S1). Therefore, we used Spearman correlation coefficient values for the analysis of the relationships between lethality values and the clinical trial and approval numbers, and the network weight values (Table 3). The significance of the correlations was determined by a permutation based algorithm [15]. We also analyzed the dependence of the clinical trials, FDA approval, weighted degree and specific drug percentage values to the lethality values by linear regression (Figure S2, S6 and S7). The significance of the linear regression was determined by the p-values of the F-test. Multiple r^2^ values of the linear fit are also provided. Linear fit parameters and their 95% confidence intervals are in the supplementary figure legends.

## Supporting Information

Text S1 (0.02 MB DOC)

Figure S1 FDA drug approval and clinical drug trial numbers. Number of FDA approvals (A) clinical trials (B) for 23 cancers in this study.
(0.04 MB PDF)

Figure S2 FDA approval and clinical trial numbers vs. lethality values. FDA approval number values are plotted against global lethality ratio for (A) 20 cancers (r2 = 0.17, p = 0.07, equation: y = 44.70x +4.28, 95% confidence intervals: (−3.59, 92.99), (0.70, 7.86)), (B) the cancers except lung cancer (r2 = 0.20, p = 0.05, equation: y = 127.67x +1.84, 95% confidence intervals: (−1.60, 256.94), (−3.14, 6.81)). FDA approval number values are plotted against local lethality ratio for (C) 20 cancers (r2 = 0.00, p = 0.99, equation: y = −0.04x +6.27, 95% confidence intervals: (−11.71, 11.62), (0.38, 12.16)), (D) the cancers except pancreatic, liver and esophagus cancers (r2 = 0.09, p = 0.23, equation: y = 9.66x +3.72, 95% confidence intervals: (−6.84, 26.16), (−2.96, 10.40)). Clinical trial number values are plotted against global lethality ratio for (E) 20 cancers (r2 = 0.25, p = 0.03, equation: y = 374.90x +32.31, 95% confidence intervals: (52.26, 697.54), (8.39, 56.24)), (F) the cancers except lung cancer (r2 = 0.21, p = 0.05, equation: y = 877.21x +17.53, 95% confidence intervals: (3.98, 1750.45), (−16.06, 51.13)). Clinical trial number values are plotted against local lethality ratio for (G) 20 cancers (r2 = 0.03, p = 0.49, equation: y = 27.36x +37.19, 95% confidence intervals: (−53.25, 107.97), (−3.53, 77.91)), (H) the cancers except pancreatic, liver and esophagus cancers (r2 = 0.20, p = 0.07, equation: y = 101.04x +17.80, 95% confidence intervals: (−10.51, 212.60), (−27.32, 62.93)). Lung cancer is shown as an open triangle and pancreatic, liver, esophagus cancers are shown as open circles.
(0.04 MB PDF)

Figure S3 FDA cancer networks of previous years. FDA cancer network of (A) 1949, (B) 1986, (C) 1991, (D) 1993, (E) 1997, (F) 1998, (G) 2000, (H) 2003, (I) 2004, (J) 2005, (K) 2006.
(0.87 MB PDF)

Figure S4 Time dependent characteristics of the FDA approvals and FDA cancer network. (A) Number of cancers in the network from 1980–2008, (B) Number of FDA approvals from 1980–2008, (C) Average weight of the network from 1980–2008, (D) Number of components of the network from 1980–2008.
(0.04 MB PDF)

Figure S5 Clinical trial cancer network.
(0.09 MB PDF)

Figure S6 FDA and clinical trial cancer network weight values vs. lethality values. FDA cancer network weight values are plotted against global lethality ratio for (A) 20 cancers (r2 = 0.18, p = 0.07, equation: y = 2.53x +0.56, 95% confidence intervals: (−0.18, 5.23), (0.36, 0.76)), (B) the cancers except lung cancer (r2 = 0.01, p = 0.69, equation: y = 1.47x +0.59, 95% confidence intervals: (−6.18, 9.12), (0.30, 0.88)). FDA cancer network weight values are plotted against local lethality ratio for (C) 20 cancers (r2 = 0.01, p = 0.78, equation: y = 0.09x +0.63, 95% confidence intervals: (−0.56, 0.74), (0.30, 0.96)), (D) the cancers except pancreatic, liver and esophagus cancers (r2 = 0.35, p = 0.01, equation: y = 0.96x +0.40, 95% confidence intervals: (0.23, 1.69), (0.11, 0.70)). Clinical trial cancer network weight values are plotted against global lethality ratio for (E) 20 cancers (r2 = 0.15, p = 0.09, equation: y = 10.02x +5.26, 95% confidence intervals: (−1.66, 21.70), (4.40, 6.13)), (F) 20 cancers except lung cancer (r2 = 0.14, p = 0.12, equation: y = 24.85x +4.83, 95% confidence intervals: (−7.28, 56.98), (3.59, 6.06)). Clinical trial cancer network weight values are plotted against local lethality ratio for (G) 20 cancers (r2 = 0.26, p = 0.02, equation: y = 2.87x +4.48, 95% confidence intervals: (0.47, 5.27), (3.27, 5.69)), (H) the cancers except pancreatic, liver and esophagus cancers (r2 = 0.20, p = 0.07, equation: y = 3.35x +4.36, 95% confidence intervals: (−0.37, 7.07), (2.85, 5.86)). Lung cancer is shown as an open triangle and pancreatic, liver, esophagus cancers are shown as open circles.
(0.04 MB PDF)

Figure S7 FDA specific drug percentage values vs. lethality values. FDA specific drug percentage values are plotted against global lethality ratio for (A) 20 cancers (r2 = 0.01, p = 0.71, equation: y = 0.37x +0.27, 95% confidence intervals: (−1.66, 2.40), (0.12, 0.42)), (B) the cancers except lung cancer (r2 = 0.17, p = 0.08, equation: y = 4.70x +0.14, 95% confidence intervals: (−0.55, 9.95), (−0.06, 0.34)). FDA specific drug percentage values are plotted against local lethality ratio for (C) 20 cancers (r2 = 0.14, p = 0.11, equation: y = −0.34x +0.43, 95% confidence intervals: (−0.75, 0.08), (0.22, 0.64)), (D) the cancers except pancreatic, liver and esophagus cancers (r2 = 0.00, p = 0.86, equation: y = −0.05x +0.35, 95% confidence intervals: (−0.66, 0.56), (0.11, 0.60)). Lung cancer is shown as an open triangle and pancreatic, liver, esophagus cancers are shown as open circles.
(0.04 MB PDF)

Figure S8 Drug/mutation target-based cancer networks. (A) Drug target-based cancer network, (B) Mutation target-based cancer network.
(0.38 MB PDF)

Figure S9 Cluster dendogram of cancer types based on global and local lethality values.
(0.11 MB TIF)

Dataset S1 Drug and cancer-type association with a year tag, based on FDA labels
(0.04 MB XLS)

Dataset S2 Phase 1, 2, and 3 clinical drug trials for cancer, completed by 2009
(0.39 MB XLS)

Dataset S3 Drug and cancer-type association with a year tag of start date, based on clinical trials
(0.14 MB XLS)

Dataset S4 Targets of FDA approved cancer drugs
(0.04 MB XLS)

Dataset S5 FDA drug targets of different cancer types
(0.03 MB XLS)

Dataset S6 Mutation targets of different cancer types
(0.03 MB XLS)

Table S1 Global and local lethality ratio values for different cancers from 2001 to 2007
(0.08 MB DOC)

Table S2 Correlation values of weighted degree, approval number values with global and local lethality values for 2001–2007
(0.06 MB DOC)

Table S3 Weight of the edges for the cancer networks based on FDA approvals and clinical trials (Weights of cancer pairs with at least one interaction in one of the two networks are given for both FDA and clinical trial cancer networks.)
(0.38 MB DOC)

Table S4 Weighted degree values and p-values for FDA cancer network from 2000 to 2007
(0.11 MB DOC)

Table S5 Clinical trial numbers along with distinct drug number and specific drug number for clinical trials
(0.05 MB DOC)

Table S6 Cancer type pairs based on FDA drug targets, together with weight of the edges
(0.17 MB DOC)

Table S7 Cancer type pairs based on mutation targets, together with weight of the edges
(0.11 MB DOC)

Table S8 Comparison of mutation target-based and drug target-based weight values of cancer pairs
(0.21 MB DOC)
