# Supplementary material for: Cancer-Drug Associations: A Complex System
Source: PLoS One. 2010 Apr 2;5(4):e10031. doi: 10.1371/journal.pone.0010031 (PMC2848862; doi:10.1371/journal.pone.0010031)
Supplement: Text S1 — (0.02 MB DOC) [file pone.0010031.s001.doc]

**Predicted values from linear regression**

Linear fits suggest that the values of the lethal cancers affect the linear relationship, therefore we estimated the values based on the equation with the other cancers. Based on the linear regression of the local lethality ratio with the FDA cancer network weighted degree values (y = 0.96x + 0.40), pancreatic, liver and esophagus cancers should have been expected to have a weighted degree of around 1.2; however, they have a weighted degree of around 0.07-0.5.

**Analysis of cancer networks in previous years**

Using the date at which a drug was approved by the FDA, we analyzed the cancer networks at earlier time points. Based on the first cancer drug, mechlorethamine, approved in 1949, leukemia, lymphoma and lung cancer can be connected in a FDA cancer network (Fig. S3). These 3 cancers for a long time were the only cancers for which a drug was approved by the FDA. This initial network began to grow as drugs for other cancers were approved by the FDA, joining the network from 1986 onwards (Figs. S3 and S4). We defined the average weight as the average of the weights of all the edges of a network (0 weights are not included). The average weight of the FDA cancer networks was calculated to capture the changes in how the drugs are shared between the different cancers over the years (Fig. S4). The average weight of the FDA cancer network was at the lowest in 1997, suggesting that the sharing of drugs between cancer types was not very high in 1997 as compared to the other years. Sharing of drugs between cancer types increased between 1998 and 2004. We also determined the component numbers of the FDA cancer networks. The network consisted of a single component except between 1991-1998 when the network contained 2 components (Figs. S3 and S4). There have been only two cases where a drug led to a jumping growth as new drugs were approved for cancers (Fig. S3). Pamidronate was approved for breast cancer and myeloma in 1991, making a connection between the two cancers, separate from the other cancers. Paclitaxel was approved for skin cancer in 1997, making a connection to ovarian cancer, causing another jump in the growth of the network. Some FDA approvals could be terminated later, however, this analysis is only concerned with the drugs which were not terminated by 2009. The time of completion of the clinical trials is not available for the majority of the trials, therefore a time dependent study of the clinical trial-based cancer networks was not performed.

**Normal distribution test**

Shapiro-Wilk test is used to analyze whether the FDA drug approval numbers, the specific FDA drug percentage values, the clinical trial numbers, the FDA and clinical trial cancer network weighted degree values, and the global and local lethality ratio values are normally distributed. If p-values obtained from this test are lower than 0.05, the null hypothesis that data are normally distributed is rejected. p-values for the 23 cancers and the cancers minus head and neck cancer, mesothelioma and sarcoma are, respectively, 4.775e-05 and 0.0003658 for FDA approval numbers, 0.002419 and 0.02321 for specific FDA drug percentage values, 0.001606 and 0.003714 for clinical trial numbers, 0.1582 and 0.3365 for FDA cancer network weighted degree values, 0.07316 and 0.0341 for clinical trial cancer network weighted degree values. p-value is 1.075e-06 for the global lethality values, and 0.07739 for the local lethality values.
