# Supplementary figures and images for: Cancer-Drug Associations: A Complex System
Source: PLoS One. 2010 Apr 2;5(4):e10031. doi: 10.1371/journal.pone.0010031 (PMC2848862; doi:10.1371/journal.pone.0010031)

A

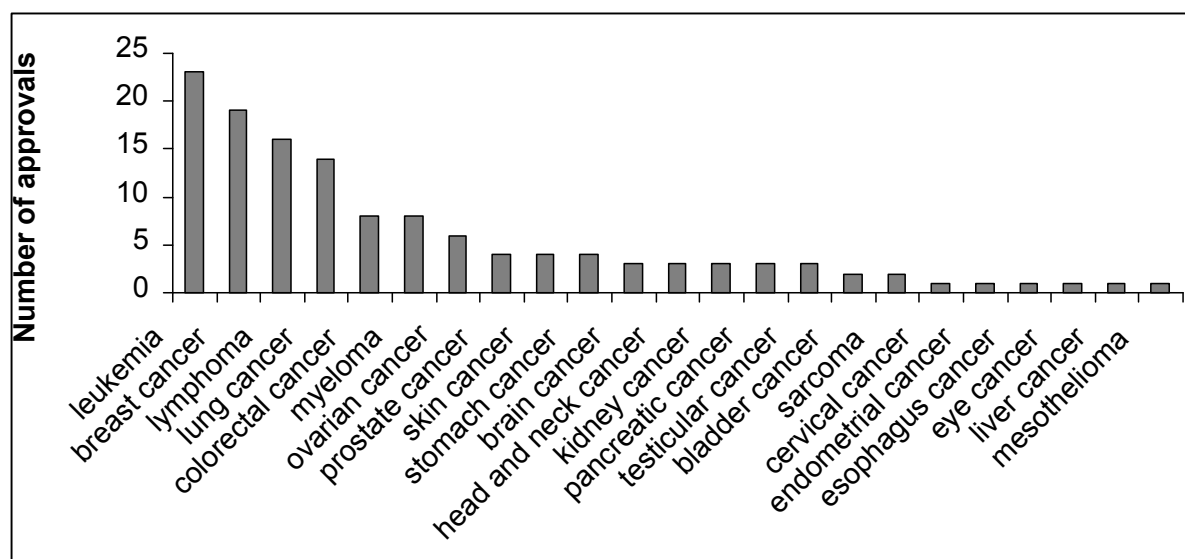

B

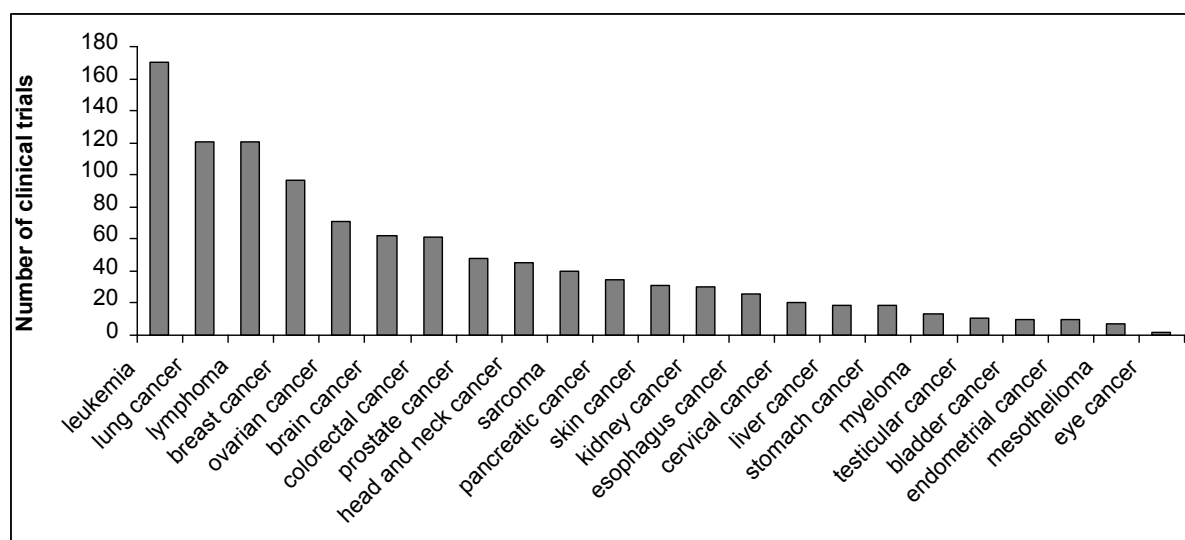

Supplement: Figure S1 — FDA drug approval and clinical drug trial numbers. Number of FDA approvals (A) clinical trials (B) for 23 cancers in this study. (0.04 MB PDF) [file pone.0010031.s002.pdf]

A

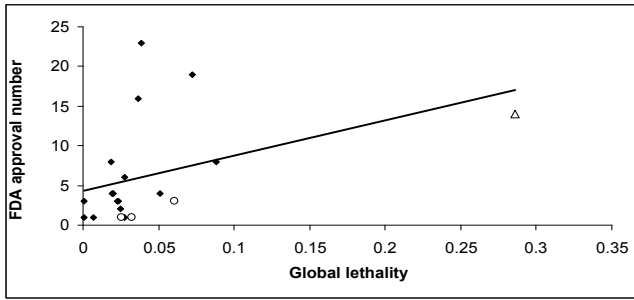

E

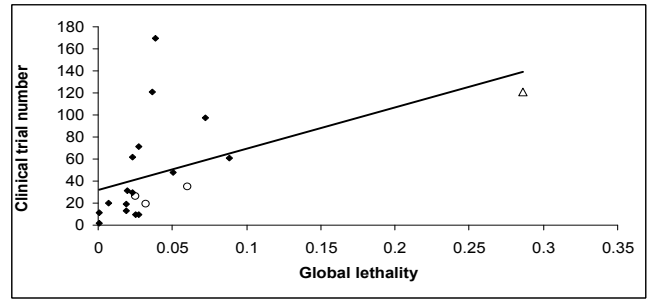

B

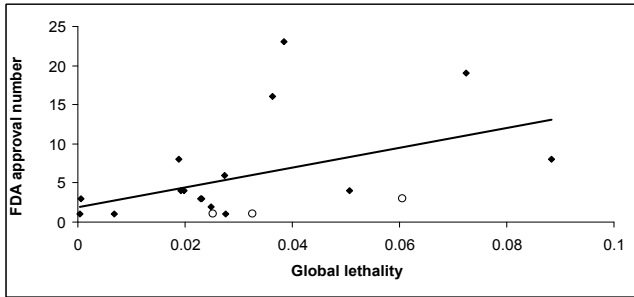

F

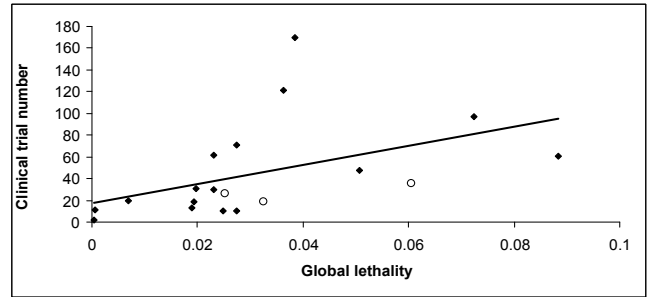

C

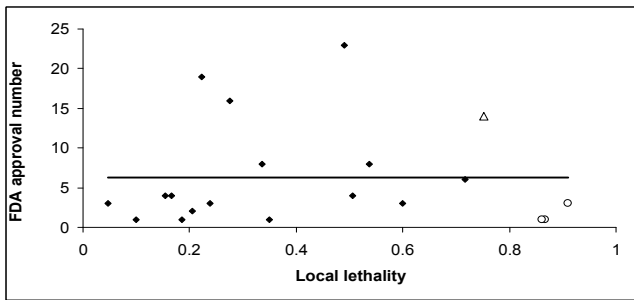

G

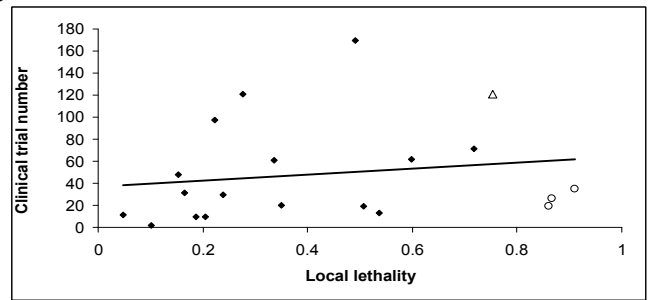

D

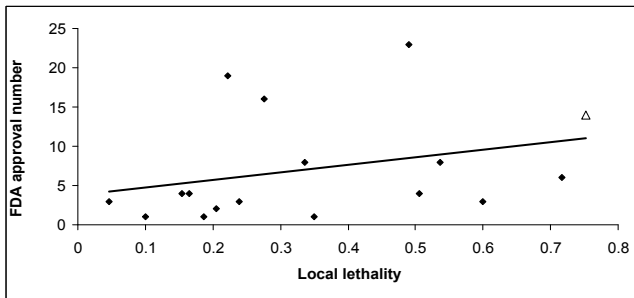

H

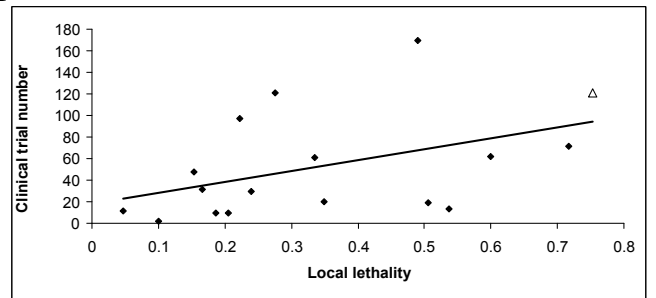

Supplement: Figure S2 — FDA approval and clinical trial numbers vs. lethality values. FDA approval number values are plotted against global lethality ratio for (A) 20 cancers (r2 = 0.17, p = 0.07, equation: y = 44.70x +4.28, 95% confidence intervals: (−3.59, 92.99), (0.70, 7.86)), (B) the cancers except lung cancer (r2 = 0.20, p = 0.05, equation: y = 127.67x +1.84, 95% confidence intervals: (−1.60, 256.94), (−3.14, 6.81)). FDA approval number values are plotted against local lethality ratio for (C) 20 cancers (r2 = 0.00, p = 0.99, equation: y = −0.04x +6.27, 95% confidence intervals: (−11.71, 11.62), (0.38, 12.16)), (D) the cancers except pancreatic, liver and esophagus cancers (r2 = 0.09, p = 0.23, equation: y = 9.66x +3.72, 95% confidence intervals: (−6.84, 26.16), (−2.96, 10.40)). Clinical trial number values are plotted against global lethality ratio for (E) 20 cancers (r2 = 0.25, p = 0.03, equation: y = 374.90x +32.31, 95% confidence intervals: (52.26, 697.54), (8.39, 56.24)), (F) the cancers except lung cancer (r2 = 0.21, p = 0.05, equation: y = 877.21x +17.53, 95% confidence intervals: (3.98, 1750.45), (−16.06, 51.13)). Clinical trial number values are plotted against local lethality ratio for (G) 20 cancers (r2 = 0.03, p = 0.49, equation: y = 27.36x +37.19, 95% confidence intervals: (−53.25, 107.97), (−3.53, 77.91)), (H) the cancers except pancreatic, liver and esophagus cancers (r2 = 0.20, p = 0.07, equation: y = 101.04x +17.80, 95% confidence intervals: (−10.51, 212.60), (−27.32, 62.93)). Lung cancer is shown as an open triangle and pancreatic, liver, esophagus cancers are shown as open circles. (0.04 MB PDF) [file pone.0010031.s003.pdf]

A

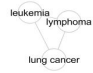

B

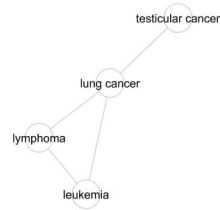

C

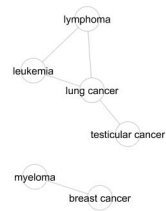

D

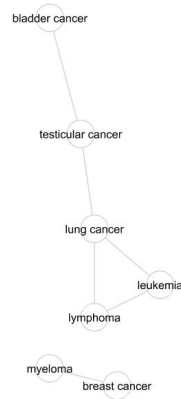

E

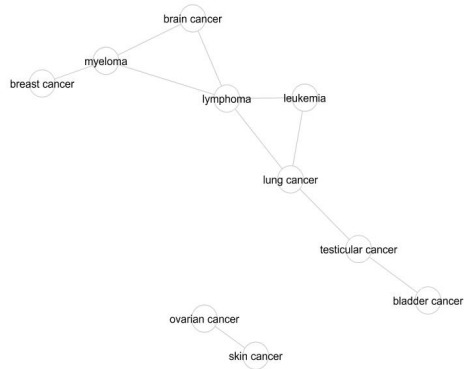

F

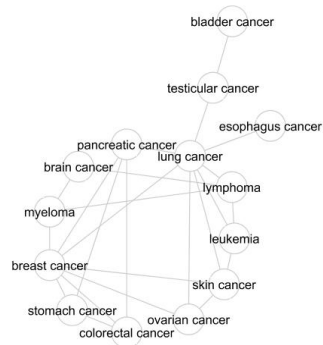

G

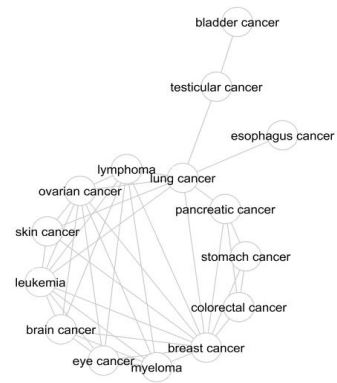

H

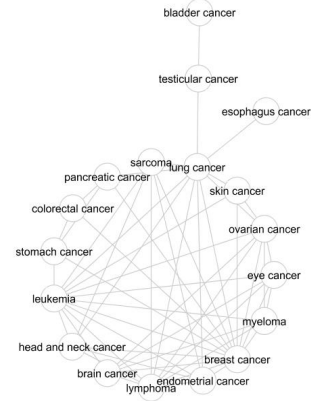

I

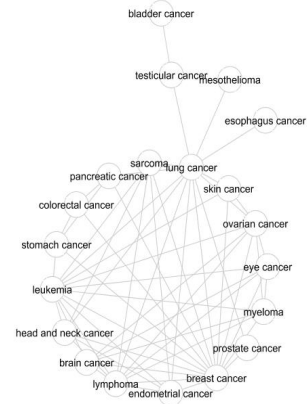

J

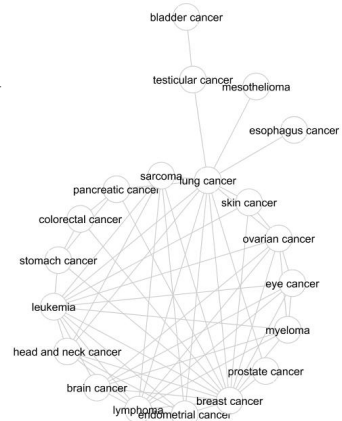

K

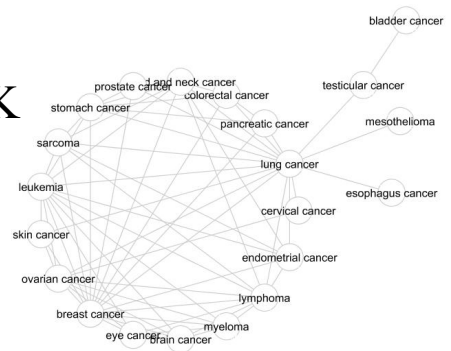

Supplement: Figure S3 — FDA cancer networks of previous years. FDA cancer network of (A) 1949, (B) 1986, (C) 1991, (D) 1993, (E) 1997, (F) 1998, (G) 2000, (H) 2003, (I) 2004, (J) 2005, (K) 2006. (0.87 MB PDF) [file pone.0010031.s004.pdf]

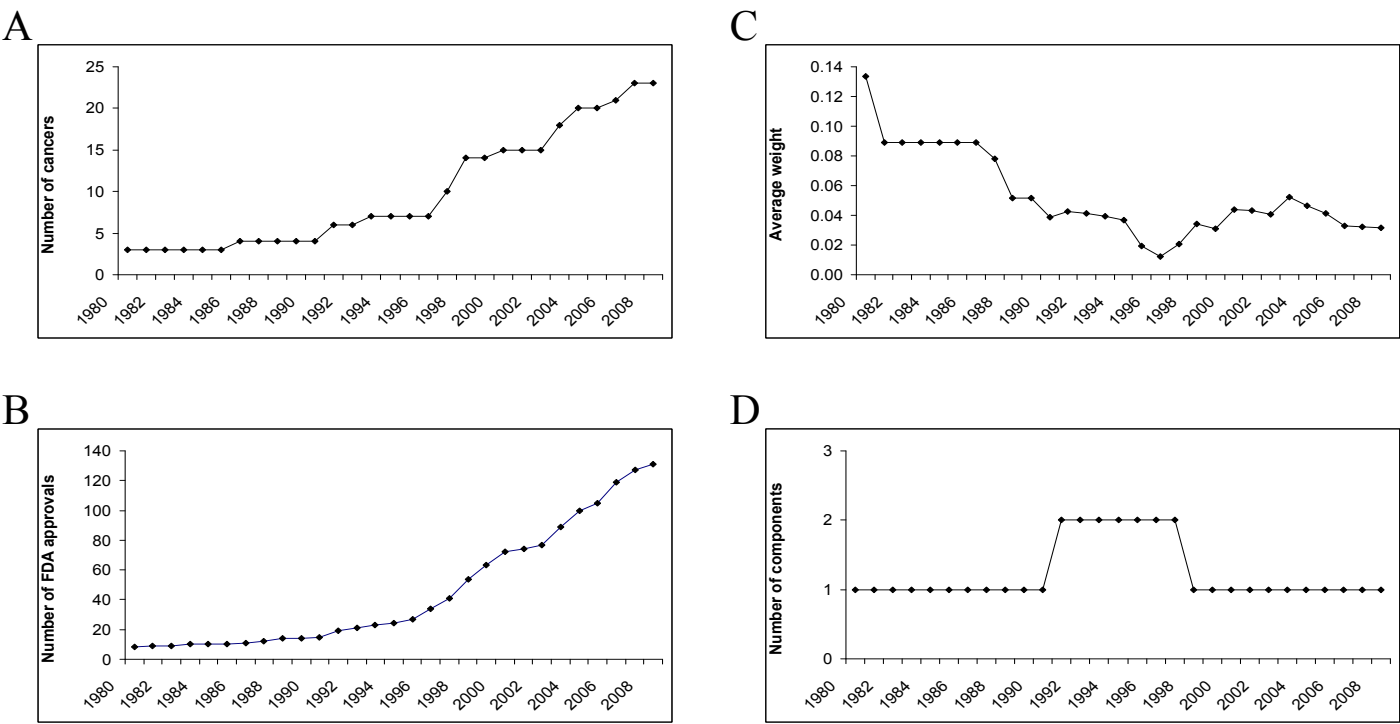

Supplement: Figure S4 — Time dependent characteristics of the FDA approvals and FDA cancer network. (A) Number of cancers in the network from 1980–2008, (B) Number of FDA approvals from 1980–2008, (C) Average weight of the network from 1980–2008, (D) Number of components of the network from 1980–2008. (0.04 MB PDF) [file pone.0010031.s005.pdf]

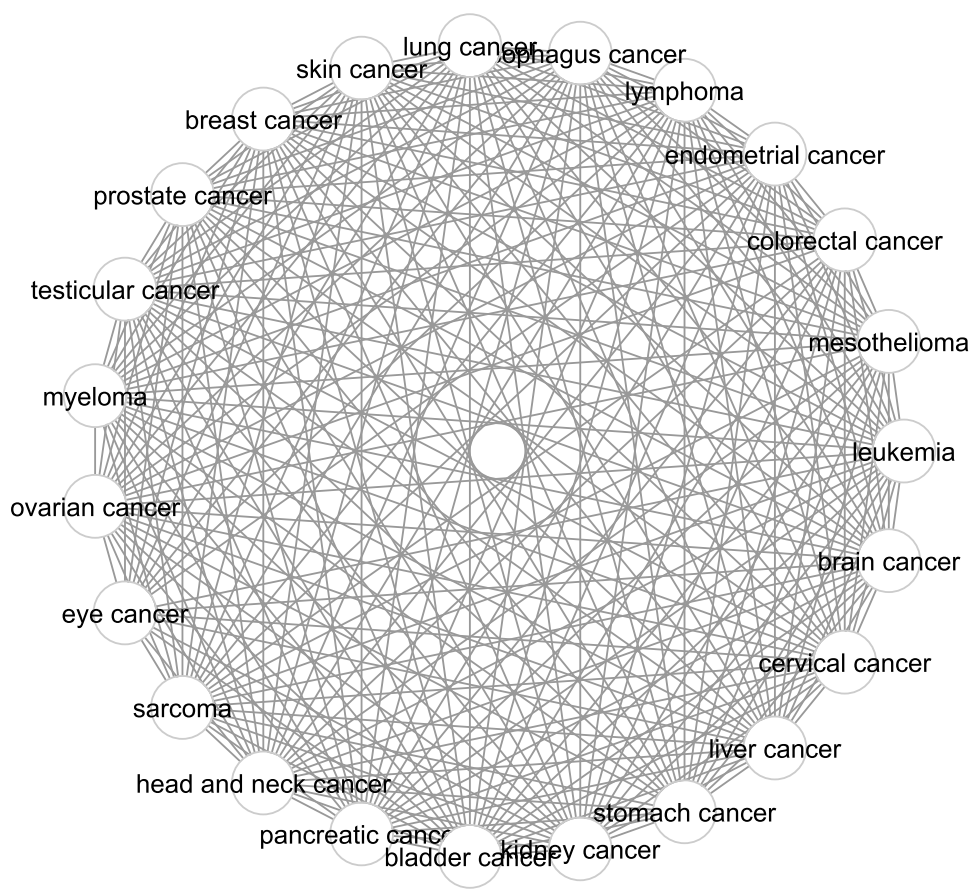

Supplement: Figure S5 — Clinical trial cancer network. (0.09 MB PDF) [file pone.0010031.s006.pdf]

A

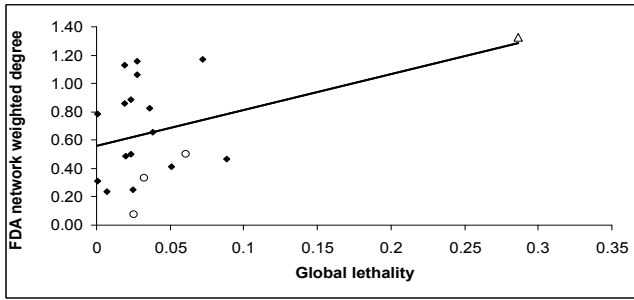

E

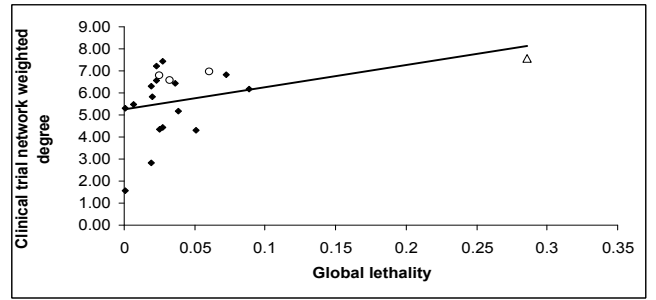

B

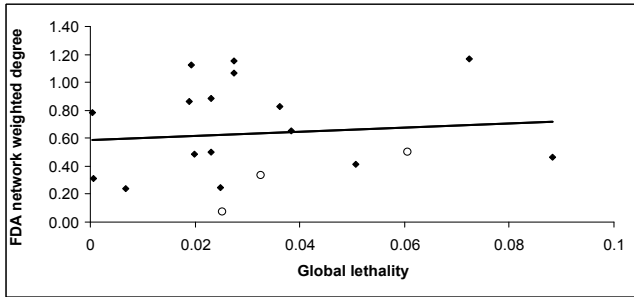

F

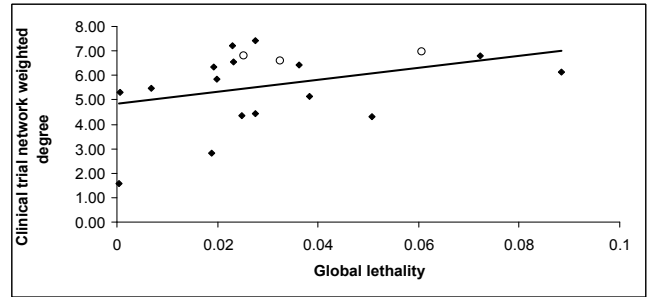

C

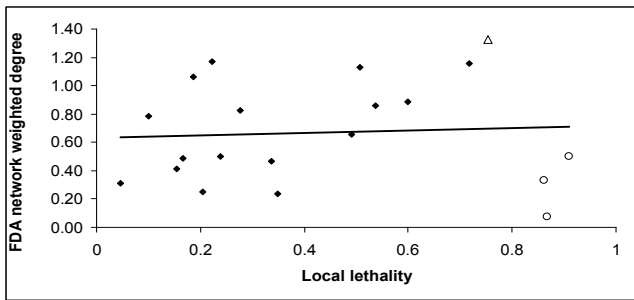

G

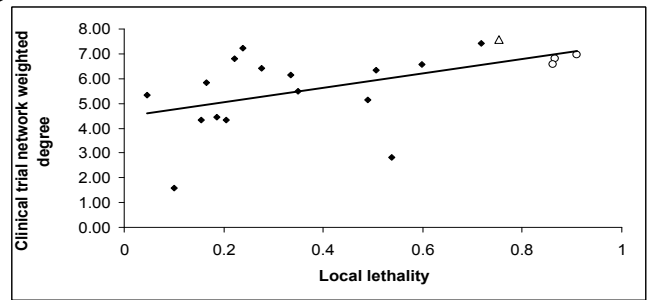

D

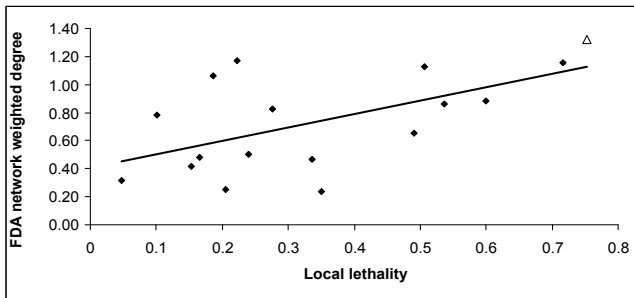

H

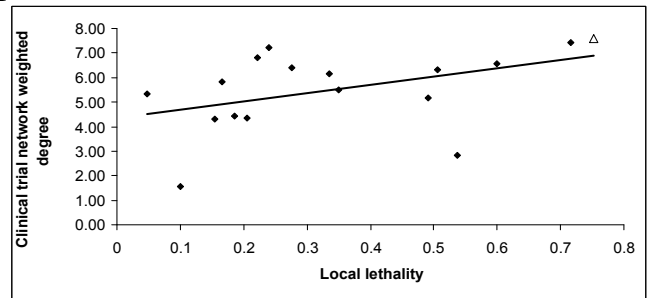

Supplement: Figure S6 — FDA and clinical trial cancer network weight values vs. lethality values. FDA cancer network weight values are plotted against global lethality ratio for (A) 20 cancers (r2 = 0.18, p = 0.07, equation: y = 2.53x +0.56, 95% confidence intervals: (−0.18, 5.23), (0.36, 0.76)), (B) the cancers except lung cancer (r2 = 0.01, p = 0.69, equation: y = 1.47x +0.59, 95% confidence intervals: (−6.18, 9.12), (0.30, 0.88)). FDA cancer network weight values are plotted against local lethality ratio for (C) 20 cancers (r2 = 0.01, p = 0.78, equation: y = 0.09x +0.63, 95% confidence intervals: (−0.56, 0.74), (0.30, 0.96)), (D) the cancers except pancreatic, liver and esophagus cancers (r2 = 0.35, p = 0.01, equation: y = 0.96x +0.40, 95% confidence intervals: (0.23, 1.69), (0.11, 0.70)). Clinical trial cancer network weight values are plotted against global lethality ratio for (E) 20 cancers (r2 = 0.15, p = 0.09, equation: y = 10.02x +5.26, 95% confidence intervals: (−1.66, 21.70), (4.40, 6.13)), (F) 20 cancers except lung cancer (r2 = 0.14, p = 0.12, equation: y = 24.85x +4.83, 95% confidence intervals: (−7.28, 56.98), (3.59, 6.06)). Clinical trial cancer network weight values are plotted against local lethality ratio for (G) 20 cancers (r2 = 0.26, p = 0.02, equation: y = 2.87x +4.48, 95% confidence intervals: (0.47, 5.27), (3.27, 5.69)), (H) the cancers except pancreatic, liver and esophagus cancers (r2 = 0.20, p = 0.07, equation: y = 3.35x +4.36, 95% confidence intervals: (−0.37, 7.07), (2.85, 5.86)). Lung cancer is shown as an open triangle and pancreatic, liver, esophagus cancers are shown as open circles. (0.04 MB PDF) [file pone.0010031.s007.pdf]

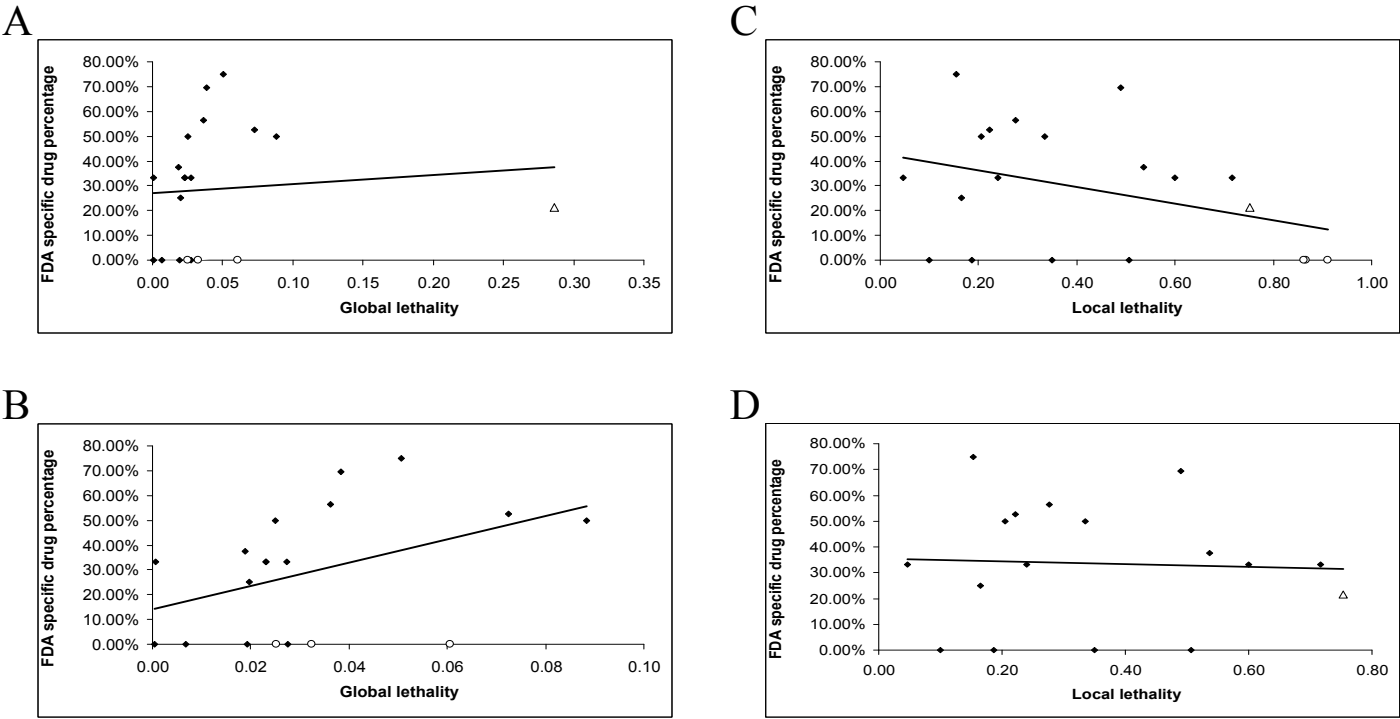

Supplement: Figure S7 — FDA specific drug percentage values vs. lethality values. FDA specific drug percentage values are plotted against global lethality ratio for (A) 20 cancers (r2 = 0.01, p = 0.71, equation: y = 0.37x +0.27, 95% confidence intervals: (−1.66, 2.40), (0.12, 0.42)), (B) the cancers except lung cancer (r2 = 0.17, p = 0.08, equation: y = 4.70x +0.14, 95% confidence intervals: (−0.55, 9.95), (−0.06, 0.34)). FDA specific drug percentage values are plotted against local lethality ratio for (C) 20 cancers (r2 = 0.14, p = 0.11, equation: y = −0.34x +0.43, 95% confidence intervals: (−0.75, 0.08), (0.22, 0.64)), (D) the cancers except pancreatic, liver and esophagus cancers (r2 = 0.00, p = 0.86, equation: y = −0.05x +0.35, 95% confidence intervals: (−0.66, 0.56), (0.11, 0.60)). Lung cancer is shown as an open triangle and pancreatic, liver, esophagus cancers are shown as open circles. (0.04 MB PDF) [file pone.0010031.s008.pdf]

A

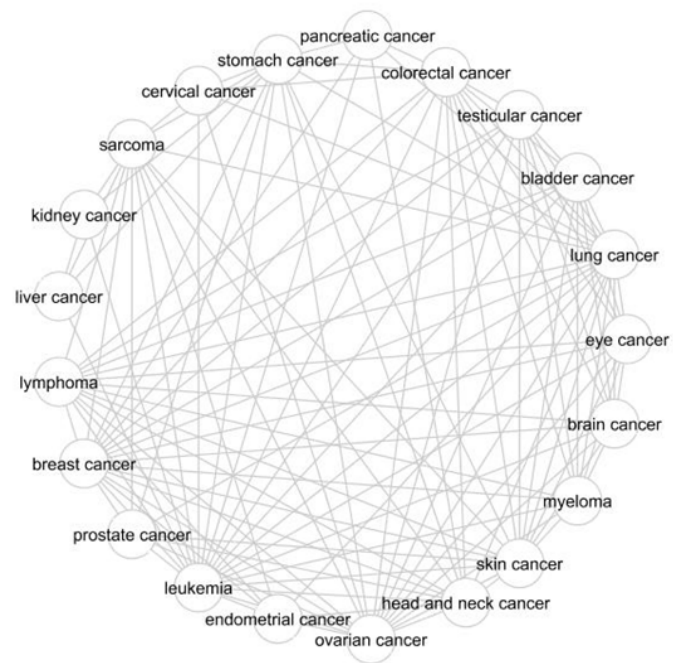

B

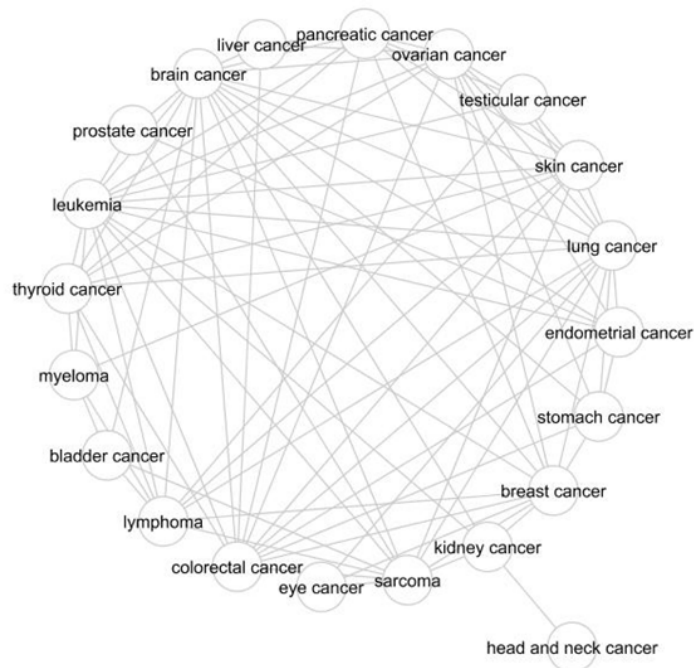

Supplement: Figure S8 — Drug/mutation target-based cancer networks. (A) Drug target-based cancer network, (B) Mutation target-based cancer network. (0.38 MB PDF) [file pone.0010031.s009.pdf]

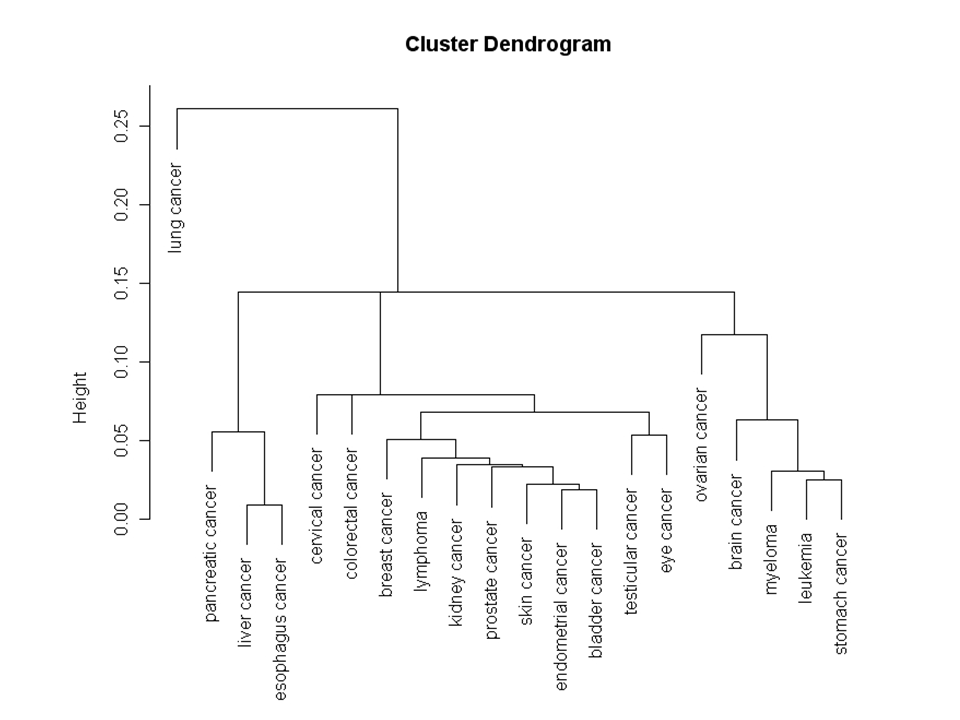

Supplement: Figure S9 — Cluster dendogram of cancer types based on global and local lethality values. (0.11 MB TIF) [file pone.0010031.s010.tif]
