# Supplementary material for: Cancer-Drug Associations: A Complex System
Source: PLoS One. 2010 Apr 2;5(4):e10031. doi: 10.1371/journal.pone.0010031 (PMC2848862; doi:10.1371/journal.pone.0010031)
Supplement: Table S1 — Global and local lethality ratio values for different cancers from 2001 to 2007 (0.08 MB DOC) [file pone.0010031.s017.doc]

**Table S1.** Global and local lethality ratio values for different cancers from 2001 to 2007.

| **Cancer type** | **2001 Global lethality ratio** | **2001 Local lethality ratio** | **2002 Global lethality ratio** | **2002 Local lethality ratio** | **2003 Global lethality ratio** | **2003 Local lethality ratio** | **2004 Global lethality ratio** | **2004 Local lethality ratio** | **2005 Global lethality ratio** | **2005 Local lethality ratio** | **2006 Global lethality ratio** | **2006 Local lethality ratio** | **2007 Global lethality ratio** | **2007 Local lethality ratio** |
| --- | --- | --- | --- | --- | --- | --- | --- | --- | --- | --- | --- | --- | --- | --- |
| lung cancer | 0.284 | 0.929 | 0.280 | 0.914 | 0.283 | 0.914 | 0.285 | 0.923 | 0.287 | 0.947 | 0.288 | 0.931 | 0.287 | 0.752 |
| colorectal cancer | 0.103 | 0.360 | 0.102 | 0.328 | 0.103 | 0.680 | 0.101 | 0.267 | 0.099 | 0.268 | 0.098 | 0.259 | 0.093 | 0.232 |
| breast cancer | 0.150 | 0.209 | 0.148 | 0.195 | 0.147 | 0.188 | 0.147 | 0.186 | 0.147 | 0.191 | 0.150 | 0.192 | 0.150 | 0.227 |
| pancreatic cancer | 0.052 | 0.990 | 0.054 | 0.980 | 0.054 | 0.977 | 0.056 | 0.981 | 0.056 | 0.988 | 0.057 | 0.958 | 0.060 | 0.898 |
| prostate cancer | 0.110 | 0.159 | 0.106 | 0.160 | 0.101 | 0.131 | 0.103 | 0.130 | 0.103 | 0.131 | 0.094 | 0.117 | 0.093 | 0.124 |
| leukemia | 0.039 | 0.682 | 0.039 | 0.704 | 0.039 | 0.716 | 0.041 | 0.697 | 0.040 | 0.648 | 0.039 | 0.635 | 0.039 | 0.492 |
| lymphoma | 0.050 | 0.434 | 0.047 | 0.424 | 0.044 | 0.405 | 0.037 | 0.333 | 0.036 | 0.323 | 0.036 | 0.305 | 0.035 | 0.276 |
| liver cancer | 0.026 | 0.870 | 0.026 | 0.849 | 0.026 | 0.832 | 0.025 | 0.754 | 0.027 | 0.879 | 0.029 | 0.875 | 0.030 | 0.876 |
| endometrial cancer | 0.025 | 0.172 | 0.025 | 0.168 | 0.025 | 0.170 | 0.026 | 0.176 | 0.027 | 0.179 | 0.027 | 0.178 | 0.027 | 0.189 |
| ovarian cancer | 0.052 | 0.594 | 0.052 | 0.597 | 0.053 | 0.563 | 0.059 | 0.629 | 0.059 | 0.729 | 0.056 | 0.759 | 0.057 | 0.681 |
| esophagus cancer | 0.023 | 0.947 | 0.023 | 0.962 | 0.023 | 0.935 | 0.024 | 0.933 | 0.024 | 0.935 | 0.024 | 0.946 | 0.025 | 0.896 |
| bladder cancer | 0.022 | 0.228 | 0.023 | 0.223 | 0.023 | 0.218 | 0.023 | 0.211 | 0.023 | 0.208 | 0.023 | 0.213 | 0.025 | 0.205 |
| brain cancer | 0.024 | 0.762 | 0.024 | 0.771 | 0.024 | 0.716 | 0.023 | 0.690 | 0.022 | 0.690 | 0.023 | 0.681 | 0.023 | 0.621 |
| kidney cancer | 0.022 | 0.393 | 0.021 | 0.365 | 0.021 | 0.373 | 0.022 | 0.349 | 0.022 | 0.350 | 0.023 | 0.330 | 0.023 | 0.252 |
| skin cancer | 0.018 | 0.174 | 0.017 | 0.165 | 0.018 | 0.167 | 0.018 | 0.173 | 0.019 | 0.160 | 0.019 | 0.156 | 0.019 | 0.167 |
| stomach cancer | 0.023 | 0.590 | 0.022 | 0.574 | 0.022 | 0.540 | 0.021 | 0.519 | 0.020 | 0.528 | 0.020 | 0.513 | 0.020 | 0.527 |
| myeloma | 0.020 | 0.778 | 0.020 | 0.740 | 0.020 | 0.747 | 0.020 | 0.725 | 0.020 | 0.707 | 0.020 | 0.683 | 0.019 | 0.542 |
| cervical cancer | 0.017 | 0.341 | 0.015 | 0.315 | 0.015 | 0.336 | 0.014 | 0.371 | 0.014 | 0.358 | 0.014 | 0.381 | 0.014 | 0.329 |
| testicular cancer | 0.001 | 0.056 | 0.001 | 0.053 | 0.001 | 0.053 | 0.001 | 0.040 | 0.001 | 0.049 | 0.001 | 0.045 | 0.001 | 0.048 |
| eye cancer | 0.000 | 0.095 | 0.000 | 0.091 | 0.000 | 0.091 | 0.000 | 0.086 | 0.000 | 0.108 | 0.000 | 0.097 | 0.000 | 0.094 |
