# Supplementary material for: Cancer-Drug Associations: A Complex System
Source: PLoS One. 2010 Apr 2;5(4):e10031. doi: 10.1371/journal.pone.0010031 (PMC2848862; doi:10.1371/journal.pone.0010031)
Supplement: Table S2 — Correlation values of weighted degree, approval number values with global and local lethality values for 2001–2007 (0.06 MB DOC) [file pone.0010031.s018.doc]

**Table S2.** Correlation values of weighted degree, approval number values with global and local lethality values for 2001-2007.

|  | FDA approval number | | | | FDA cancer network weighted degree | | | |  |
| --- | --- | --- | --- | --- | --- | --- | --- | --- | --- |
|  | Global lethality | | Local lethality | | Global lethality | | Local lethality | |  |
|  | All cancers | All cancers except lung cancer | All cancers | All cancers except pancreatic, liver and esophagus cancers | All cancers | All cancers except lung cancer | All cancers | All cancers except pancreatic, liver and esophagus cancers |  |
| 2001 | 0.507883 | 0.440047 | 0.062646 | 0.40361 | -0.01325 | -0.01431 | 0.267647 | 0.481319 | Spearman statistic |
|  | 0.044594 | 0.100702 | 0.817717 | 0.152398 | 0.961142 | 0.959628 | 0.312408 | 0.082451 | Spearman p-value |
| 2002 | 0.493703 | 0.434419 | 0.078756 | 0.425737 | 0.010302 | -0.00179 | 0.270588 | 0.485714 | Spearman statistic |
|  | 0.05195 | 0.105646 | 0.771877 | 0.129076 | 0.969796 | 0.994956 | 0.306974 | 0.079402 | Spearman p-value |
| 2003 | 0.453218 | 0.384073 | 0.203359 | 0.553168 | 0.295524 | 0.207506 | 0.144697 | 0.37891 | Spearman statistic |
|  | 0.067694 | 0.141915 | 0.433722 | 0.03244 | 0.249477 | 0.440621 | 0.57952 | 0.163683 | Spearman p-value |
| 2004 | 0.529063 | 0.454699 | 0.164304 | 0.522921 | 0.256285 | 0.133922 | 0.137255 | 0.407143 | Spearman statistic |
|  | 0.028981 | 0.076807 | 0.528597 | 0.045488 | 0.320752 | 0.620973 | 0.595315 | 0.131493 | Spearman p-value |
| 2005 | 0.549142 | 0.489158 | 0.197299 | 0.39853 | 0.273761 | 0.159509 | 0.168215 | 0.358824 | Spearman statistic |
|  | 0.018258 | 0.046291 | 0.432609 | 0.126272 | 0.271658 | 0.540859 | 0.500542 | 0.170818 | Spearman p-value |
| 2006 | 0.525673 | 0.4698 | 0.174395 | 0.389871 | 0.414946 | 0.313503 | 0.124561 | 0.330882 | Spearman statistic |
|  | 0.020802 | 0.049159 | 0.475188 | 0.121863 | 0.077301 | 0.205218 | 0.607889 | 0.192679 | Spearman p-value |
| 2007 | 0.479393 | 0.415906 | 0.072584 | 0.449279 | 0.301958 | 0.185413 | 0.171429 | 0.573529 | Spearman statistic |
|  | 0.032453 | 0.076549 | 0.761048 | 0.070419 | 0.195699 | 0.447281 | 0.466237 | 0.017639 | Spearman p-value |
