# Supplementary material for: Cancer-Drug Associations: A Complex System
Source: PLoS One. 2010 Apr 2;5(4):e10031. doi: 10.1371/journal.pone.0010031 (PMC2848862; doi:10.1371/journal.pone.0010031)
Supplement: Table S3 — Weight of the edges for the cancer networks based on FDA approvals and clinical trials (Weights of cancer pairs with at least one interaction in one of the two networks are given for both FDA and clinical trial cancer networks.) (0.38 MB DOC) [file pone.0010031.s019.doc]

**Table S3.** Weight of the edges for the cancer networks based on FDA approvals and clinical trials. Weigts of cancer pairs with at least one interaction in one of the two networks are given for both FDA and clinical trial cancer networks.

| **Cancer type 1** | **Cancer type 2** | **Clinical trial cancer network weight** | **FDA cancer network weight** | **Difference** |
| --- | --- | --- | --- | --- |
| stomach cancer | esophagus cancer | 0.71 | 0.00 | 0.71 |
| head and neck cancer | kidney cancer | 0.56 | 0.00 | 0.56 |
| kidney cancer | lung cancer | 0.54 | 0.00 | 0.54 |
| ovarian cancer | head and neck cancer | 0.54 | 0.00 | 0.54 |
| leukemia | lymphoma | 0.68 | 0.15 | 0.53 |
| ovarian cancer | breast cancer | 0.61 | 0.09 | 0.53 |
| cervical cancer | esophagus cancer | 0.50 | 0.00 | 0.50 |
| head and neck cancer | brain cancer | 0.50 | 0.00 | 0.50 |
| head and neck cancer | liver cancer | 0.50 | 0.00 | 0.50 |
| stomach cancer | cervical cancer | 0.50 | 0.00 | 0.50 |
| kidney cancer | brain cancer | 0.49 | 0.00 | 0.49 |
| sarcoma | kidney cancer | 0.46 | 0.00 | 0.46 |
| liver cancer | esophagus cancer | 0.45 | 0.00 | 0.45 |
| ovarian cancer | sarcoma | 0.45 | 0.00 | 0.45 |
| stomach cancer | liver cancer | 0.45 | 0.00 | 0.45 |
| ovarian cancer | pancreatic cancer | 0.45 | 0.00 | 0.45 |
| pancreatic cancer | colorectal cancer | 0.54 | 0.10 | 0.44 |
| sarcoma | lymphoma | 0.50 | 0.06 | 0.44 |
| sarcoma | brain cancer | 0.44 | 0.00 | 0.44 |
| brain cancer | skin cancer | 0.44 | 0.00 | 0.44 |
| kidney cancer | lymphoma | 0.44 | 0.00 | 0.44 |
| stomach cancer | colorectal cancer | 0.53 | 0.09 | 0.44 |
| lymphoma | skin cancer | 0.43 | 0.00 | 0.43 |
| pancreatic cancer | head and neck cancer | 0.42 | 0.00 | 0.42 |
| pancreatic cancer | esophagus cancer | 0.42 | 0.00 | 0.42 |
| head and neck cancer | lung cancer | 0.56 | 0.13 | 0.42 |
| liver cancer | cervical cancer | 0.42 | 0.00 | 0.42 |
| ovarian cancer | kidney cancer | 0.42 | 0.00 | 0.42 |
| brain cancer | lung cancer | 0.41 | 0.00 | 0.41 |
| testicular cancer | sarcoma | 0.41 | 0.00 | 0.41 |
| head and neck cancer | esophagus cancer | 0.40 | 0.00 | 0.40 |
| liver cancer | lung cancer | 0.39 | 0.00 | 0.39 |
| sarcoma | lung cancer | 0.46 | 0.07 | 0.39 |
| colorectal cancer | esophagus cancer | 0.39 | 0.00 | 0.39 |
| pancreatic cancer | cervical cancer | 0.39 | 0.00 | 0.39 |
| liver cancer | endometrial cancer | 0.39 | 0.00 | 0.39 |
| ovarian cancer | liver cancer | 0.39 | 0.00 | 0.39 |
| head and neck cancer | skin cancer | 0.38 | 0.00 | 0.38 |
| sarcoma | colorectal cancer | 0.38 | 0.00 | 0.38 |
| ovarian cancer | lung cancer | 0.49 | 0.11 | 0.38 |
| kidney cancer | skin cancer | 0.37 | 0.00 | 0.37 |
| pancreatic cancer | kidney cancer | 0.37 | 0.00 | 0.37 |
| head and neck cancer | cervical cancer | 0.37 | 0.00 | 0.37 |
| sarcoma | breast cancer | 0.42 | 0.05 | 0.37 |
| pancreatic cancer | sarcoma | 0.36 | 0.00 | 0.36 |
| kidney cancer | breast cancer | 0.36 | 0.00 | 0.36 |
| kidney cancer | leukemia | 0.36 | 0.00 | 0.36 |
| ovarian cancer | colorectal cancer | 0.35 | 0.00 | 0.35 |
| ovarian cancer | esophagus cancer | 0.35 | 0.00 | 0.35 |
| cervical cancer | colorectal cancer | 0.35 | 0.00 | 0.35 |
| head and neck cancer | colorectal cancer | 0.45 | 0.10 | 0.35 |
| testicular cancer | brain cancer | 0.34 | 0.00 | 0.34 |
| kidney cancer | colorectal cancer | 0.34 | 0.00 | 0.34 |
| ovarian cancer | stomach cancer | 0.34 | 0.00 | 0.34 |
| lymphoma | lung cancer | 0.41 | 0.07 | 0.34 |
| ovarian cancer | lymphoma | 0.38 | 0.05 | 0.33 |
| cervical cancer | endometrial cancer | 0.33 | 0.00 | 0.33 |
| liver cancer | colorectal cancer | 0.33 | 0.00 | 0.33 |
| prostate cancer | pancreatic cancer | 0.33 | 0.00 | 0.33 |
| sarcoma | esophagus cancer | 0.33 | 0.00 | 0.33 |
| sarcoma | liver cancer | 0.33 | 0.00 | 0.33 |
| testicular cancer | lymphoma | 0.33 | 0.00 | 0.33 |
| head and neck cancer | lymphoma | 0.39 | 0.06 | 0.33 |
| head and neck cancer | breast cancer | 0.43 | 0.10 | 0.33 |
| brain cancer | breast cancer | 0.38 | 0.05 | 0.33 |
| esophagus cancer | breast cancer | 0.33 | 0.00 | 0.33 |
| sarcoma | skin cancer | 0.32 | 0.00 | 0.32 |
| liver cancer | brain cancer | 0.32 | 0.00 | 0.32 |
| testicular cancer | kidney cancer | 0.32 | 0.00 | 0.32 |
| pancreatic cancer | liver cancer | 0.32 | 0.00 | 0.32 |
| skin cancer | lung cancer | 0.38 | 0.06 | 0.32 |
| endometrial cancer | esophagus cancer | 0.32 | 0.00 | 0.32 |
| pancreatic cancer | breast cancer | 0.41 | 0.10 | 0.31 |
| pancreatic cancer | brain cancer | 0.31 | 0.00 | 0.31 |
| ovarian cancer | brain cancer | 0.44 | 0.13 | 0.31 |
| pancreatic cancer | skin cancer | 0.31 | 0.00 | 0.31 |
| pancreatic cancer | stomach cancer | 0.48 | 0.17 | 0.31 |
| leukemia | skin cancer | 0.35 | 0.04 | 0.31 |
| kidney cancer | cervical cancer | 0.31 | 0.00 | 0.31 |
| leukemia | lung cancer | 0.37 | 0.06 | 0.31 |
| sarcoma | bladder cancer | 0.31 | 0.00 | 0.31 |
| testicular cancer | leukemia | 0.31 | 0.00 | 0.31 |
| breast cancer | skin cancer | 0.35 | 0.05 | 0.30 |
| kidney cancer | esophagus cancer | 0.30 | 0.00 | 0.30 |
| lymphoma | breast cancer | 0.36 | 0.06 | 0.30 |
| ovarian cancer | testicular cancer | 0.30 | 0.00 | 0.30 |
| testicular cancer | skin cancer | 0.30 | 0.00 | 0.30 |
| prostate cancer | ovarian cancer | 0.30 | 0.00 | 0.30 |
| stomach cancer | endometrial cancer | 0.29 | 0.00 | 0.29 |
| pancreatic cancer | lung cancer | 0.42 | 0.13 | 0.28 |
| brain cancer | colorectal cancer | 0.28 | 0.00 | 0.28 |
| brain cancer | leukemia | 0.32 | 0.04 | 0.28 |
| ovarian cancer | leukemia | 0.31 | 0.04 | 0.28 |
| brain cancer | lymphoma | 0.40 | 0.12 | 0.28 |
| breast cancer | lung cancer | 0.46 | 0.18 | 0.28 |
| bladder cancer | stomach cancer | 0.28 | 0.00 | 0.28 |
| testicular cancer | myeloma | 0.28 | 0.00 | 0.28 |
| esophagus cancer | skin cancer | 0.28 | 0.00 | 0.28 |
| testicular cancer | breast cancer | 0.28 | 0.00 | 0.28 |
| testicular cancer | lung cancer | 0.33 | 0.06 | 0.27 |
| sarcoma | leukemia | 0.36 | 0.09 | 0.27 |
| colorectal cancer | lung cancer | 0.31 | 0.05 | 0.27 |
| colorectal cancer | mesothelioma | 0.26 | 0.00 | 0.26 |
| liver cancer | mesothelioma | 0.26 | 0.00 | 0.26 |
| liver cancer | breast cancer | 0.26 | 0.00 | 0.26 |
| pancreatic cancer | lymphoma | 0.26 | 0.00 | 0.26 |
| stomach cancer | skin cancer | 0.26 | 0.00 | 0.26 |
| bladder cancer | lung cancer | 0.25 | 0.00 | 0.25 |
| testicular cancer | esophagus cancer | 0.25 | 0.00 | 0.25 |
| testicular cancer | head and neck cancer | 0.25 | 0.00 | 0.25 |
| leukemia | breast cancer | 0.30 | 0.05 | 0.25 |
| head and neck cancer | leukemia | 0.29 | 0.04 | 0.25 |
| esophagus cancer | lung cancer | 0.31 | 0.07 | 0.24 |
| brain cancer | esophagus cancer | 0.24 | 0.00 | 0.24 |
| head and neck cancer | bladder cancer | 0.24 | 0.00 | 0.24 |
| pancreatic cancer | bladder cancer | 0.24 | 0.00 | 0.24 |
| bladder cancer | colorectal cancer | 0.24 | 0.00 | 0.24 |
| bladder cancer | esophagus cancer | 0.24 | 0.00 | 0.24 |
| prostate cancer | sarcoma | 0.24 | 0.00 | 0.24 |
| lymphoma | esophagus cancer | 0.23 | 0.00 | 0.23 |
| colorectal cancer | breast cancer | 0.36 | 0.13 | 0.23 |
| prostate cancer | lung cancer | 0.29 | 0.06 | 0.23 |
| liver cancer | skin cancer | 0.23 | 0.00 | 0.23 |
| prostate cancer | lymphoma | 0.23 | 0.00 | 0.23 |
| head and neck cancer | stomach cancer | 0.39 | 0.17 | 0.23 |
| prostate cancer | leukemia | 0.22 | 0.00 | 0.22 |
| ovarian cancer | bladder cancer | 0.22 | 0.00 | 0.22 |
| sarcoma | head and neck cancer | 0.47 | 0.25 | 0.22 |
| stomach cancer | breast cancer | 0.32 | 0.10 | 0.22 |
| prostate cancer | kidney cancer | 0.22 | 0.00 | 0.22 |
| prostate cancer | esophagus cancer | 0.22 | 0.00 | 0.22 |
| prostate cancer | liver cancer | 0.22 | 0.00 | 0.22 |
| mesothelioma | endometrial cancer | 0.21 | 0.00 | 0.21 |
| testicular cancer | eye cancer | 0.21 | 0.00 | 0.21 |
| colorectal cancer | lymphoma | 0.21 | 0.00 | 0.21 |
| liver cancer | lymphoma | 0.21 | 0.00 | 0.21 |
| sarcoma | cervical cancer | 0.21 | 0.00 | 0.21 |
| testicular cancer | pancreatic cancer | 0.21 | 0.00 | 0.21 |
| stomach cancer | lung cancer | 0.26 | 0.06 | 0.21 |
| bladder cancer | breast cancer | 0.21 | 0.00 | 0.21 |
| prostate cancer | breast cancer | 0.25 | 0.05 | 0.20 |
| bladder cancer | mesothelioma | 0.20 | 0.00 | 0.20 |
| cervical cancer | brain cancer | 0.20 | 0.00 | 0.20 |
| cervical cancer | breast cancer | 0.20 | 0.00 | 0.20 |
| kidney cancer | endometrial cancer | 0.20 | 0.00 | 0.20 |
| ovarian cancer | mesothelioma | 0.20 | 0.00 | 0.20 |
| pancreatic cancer | endometrial cancer | 0.20 | 0.00 | 0.20 |
| ovarian cancer | endometrial cancer | 0.19 | 0.00 | 0.19 |
| colorectal cancer | skin cancer | 0.19 | 0.00 | 0.19 |
| colorectal cancer | endometrial cancer | 0.19 | 0.00 | 0.19 |
| testicular cancer | cervical cancer | 0.19 | 0.00 | 0.19 |
| bladder cancer | endometrial cancer | 0.19 | 0.00 | 0.19 |
| cervical cancer | mesothelioma | 0.19 | 0.00 | 0.19 |
| stomach cancer | brain cancer | 0.19 | 0.00 | 0.19 |
| prostate cancer | endometrial cancer | 0.19 | 0.00 | 0.19 |
| mesothelioma | breast cancer | 0.18 | 0.00 | 0.18 |
| pancreatic cancer | leukemia | 0.18 | 0.00 | 0.18 |
| bladder cancer | liver cancer | 0.18 | 0.00 | 0.18 |
| bladder cancer | lymphoma | 0.18 | 0.00 | 0.18 |
| myeloma | lung cancer | 0.18 | 0.00 | 0.18 |
| prostate cancer | colorectal cancer | 0.18 | 0.00 | 0.18 |
| prostate cancer | bladder cancer | 0.18 | 0.00 | 0.18 |
| leukemia | esophagus cancer | 0.18 | 0.00 | 0.18 |
| brain cancer | endometrial cancer | 0.17 | 0.00 | 0.17 |
| head and neck cancer | mesothelioma | 0.17 | 0.00 | 0.17 |
| cervical cancer | lung cancer | 0.24 | 0.07 | 0.17 |
| prostate cancer | brain cancer | 0.17 | 0.00 | 0.17 |
| stomach cancer | lymphoma | 0.17 | 0.00 | 0.17 |
| bladder cancer | brain cancer | 0.17 | 0.00 | 0.17 |
| bladder cancer | cervical cancer | 0.17 | 0.00 | 0.17 |
| head and neck cancer | myeloma | 0.17 | 0.00 | 0.17 |
| stomach cancer | mesothelioma | 0.17 | 0.00 | 0.17 |
| kidney cancer | myeloma | 0.16 | 0.00 | 0.16 |
| pancreatic cancer | mesothelioma | 0.16 | 0.00 | 0.16 |
| myeloma | skin cancer | 0.25 | 0.09 | 0.16 |
| prostate cancer | skin cancer | 0.16 | 0.00 | 0.16 |
| prostate cancer | testicular cancer | 0.16 | 0.00 | 0.16 |
| mesothelioma | lung cancer | 0.23 | 0.07 | 0.15 |
| endometrial cancer | skin cancer | 0.15 | 0.00 | 0.15 |
| pancreatic cancer | myeloma | 0.15 | 0.00 | 0.15 |
| testicular cancer | liver cancer | 0.15 | 0.00 | 0.15 |
| liver cancer | leukemia | 0.15 | 0.00 | 0.15 |
| testicular cancer | endometrial cancer | 0.15 | 0.00 | 0.15 |
| bladder cancer | skin cancer | 0.15 | 0.00 | 0.15 |
| prostate cancer | mesothelioma | 0.15 | 0.00 | 0.15 |
| leukemia | myeloma | 0.18 | 0.03 | 0.15 |
| cervical cancer | skin cancer | 0.14 | 0.00 | 0.14 |
| mesothelioma | esophagus cancer | 0.14 | 0.00 | 0.14 |
| sarcoma | mesothelioma | 0.14 | 0.00 | 0.14 |
| sarcoma | myeloma | 0.14 | 0.00 | 0.14 |
| prostate cancer | cervical cancer | 0.13 | 0.00 | 0.13 |
| kidney cancer | mesothelioma | 0.13 | 0.00 | 0.13 |
| cervical cancer | lymphoma | 0.13 | 0.00 | 0.13 |
| endometrial cancer | breast cancer | 0.18 | 0.05 | 0.13 |
| endometrial cancer | myeloma | 0.13 | 0.00 | 0.13 |
| sarcoma | eye cancer | 0.13 | 0.00 | 0.13 |
| testicular cancer | stomach cancer | 0.13 | 0.00 | 0.13 |
| sarcoma | stomach cancer | 0.32 | 0.20 | 0.12 |
| bladder cancer | kidney cancer | 0.12 | 0.00 | 0.12 |
| bladder cancer | leukemia | 0.12 | 0.00 | 0.12 |
| bladder cancer | myeloma | 0.12 | 0.00 | 0.12 |
| esophagus cancer | eye cancer | 0.12 | 0.00 | 0.12 |
| liver cancer | eye cancer | 0.12 | 0.00 | 0.12 |
| cervical cancer | myeloma | 0.11 | 0.00 | 0.11 |
| kidney cancer | eye cancer | 0.11 | 0.00 | 0.11 |
| mesothelioma | lymphoma | 0.11 | 0.00 | 0.11 |
| testicular cancer | colorectal cancer | 0.11 | 0.00 | 0.11 |
| endometrial cancer | lung cancer | 0.18 | 0.07 | 0.11 |
| colorectal cancer | leukemia | 0.10 | 0.00 | 0.10 |
| eye cancer | lung cancer | 0.10 | 0.00 | 0.10 |
| endometrial cancer | eye cancer | 0.09 | 0.00 | 0.09 |
| esophagus cancer | myeloma | 0.09 | 0.00 | 0.09 |
| liver cancer | myeloma | 0.09 | 0.00 | 0.09 |
| bladder cancer | eye cancer | 0.08 | 0.00 | 0.08 |
| ovarian cancer | cervical cancer | 0.25 | 0.17 | 0.08 |
| stomach cancer | kidney cancer | 0.25 | 0.17 | 0.08 |
| cervical cancer | eye cancer | 0.08 | 0.00 | 0.08 |
| head and neck cancer | eye cancer | 0.07 | 0.00 | 0.07 |
| mesothelioma | skin cancer | 0.07 | 0.00 | 0.07 |
| mesothelioma | leukemia | 0.07 | 0.00 | 0.07 |
| endometrial cancer | lymphoma | 0.13 | 0.06 | 0.07 |
| cervical cancer | leukemia | 0.07 | 0.00 | 0.07 |
| prostate cancer | myeloma | 0.07 | 0.00 | 0.07 |
| brain cancer | mesothelioma | 0.06 | 0.00 | 0.06 |
| mesothelioma | myeloma | 0.06 | 0.00 | 0.06 |
| prostate cancer | head and neck cancer | 0.23 | 0.17 | 0.06 |
| kidney cancer | liver cancer | 0.39 | 0.33 | 0.05 |
| stomach cancer | leukemia | 0.09 | 0.04 | 0.05 |
| stomach cancer | myeloma | 0.05 | 0.00 | 0.05 |
| testicular cancer | mesothelioma | 0.05 | 0.00 | 0.05 |
| eye cancer | skin cancer | 0.04 | 0.00 | 0.04 |
| breast cancer | myeloma | 0.12 | 0.08 | 0.04 |
| colorectal cancer | myeloma | 0.04 | 0.00 | 0.04 |
| prostate cancer | eye cancer | 0.04 | 0.00 | 0.04 |
| leukemia | eye cancer | 0.08 | 0.04 | 0.04 |
| ovarian cancer | skin cancer | 0.28 | 0.25 | 0.03 |
| leukemia | endometrial cancer | 0.07 | 0.04 | 0.03 |
| prostate cancer | stomach cancer | 0.16 | 0.14 | 0.02 |
| lymphoma | myeloma | 0.16 | 0.14 | 0.02 |
| testicular cancer | bladder cancer | 0.26 | 0.25 | 0.01 |
| lymphoma | eye cancer | 0.07 | 0.06 | 0.01 |
| eye cancer | breast cancer | 0.05 | 0.05 | 0.00 |
| brain cancer | myeloma | 0.17 | 0.22 | -0.05 |
| ovarian cancer | myeloma | 0.10 | 0.17 | -0.06 |
| head and neck cancer | endometrial cancer | 0.25 | 0.33 | -0.08 |
| ovarian cancer | eye cancer | 0.06 | 0.17 | -0.11 |
| eye cancer | myeloma | 0.00 | 0.13 | -0.13 |
| brain cancer | eye cancer | 0.12 | 0.33 | -0.21 |
| sarcoma | endometrial cancer | 0.22 | 0.50 | -0.28 |
