# Supplementary material for: Cancer-Drug Associations: A Complex System
Source: PLoS One. 2010 Apr 2;5(4):e10031. doi: 10.1371/journal.pone.0010031 (PMC2848862; doi:10.1371/journal.pone.0010031)
Supplement: Table S4 — Weighted degree values and p-values for FDA cancer network from 2000 to 2007 (0.11 MB DOC) [file pone.0010031.s020.doc]

**Table S4. Weighted degree values and p-values for FDA cancer network from 2000 to 2007**

|  | 2000 | | 2001 | | 2002 | | 2003 | | 2004 | | 2005 | | 2006 | | 2007 | |
| --- | --- | --- | --- | --- | --- | --- | --- | --- | --- | --- | --- | --- | --- | --- | --- | --- |
|  | p-value | weighted degree | p-value | weighted degree | p-value | weighted degree | p-value | weighted degree | p-value | weighted degree | p-value | weighted degree | p-value | weighted degree | p-value | weighted degree |
| breast cancer | 0.063 | 0.944 | 0.035 | 1.013 | 0.027 | 0.936 | 0.028 | 1.192 | 0.007 | 1.269 | 0.012 | 1.250 | 0.007 | 1.327 | 0.009 | 1.139 |
| lung cancer | 0.327 | 0.774 | 0.289 | 0.771 | 0.343 | 0.763 | 0.164 | 1.049 | 0.088 | 1.092 | 0.073 | 1.158 | 0.022 | 1.315 | 0.024 | 1.296 |
| leukemia | 0.383 | 0.559 | 0.391 | 0.525 | 0.401 | 0.514 | 0.169 | 0.832 | 0.181 | 0.706 | 0.151 | 0.711 | 0.166 | 0.681 | 0.155 | 0.649 |
| lymphoma | 0.299 | 0.802 | 0.298 | 0.797 | 0.292 | 0.716 | 0.188 | 0.983 | 0.176 | 0.957 | 0.161 | 0.934 | 0.203 | 0.858 | 0.212 | 0.839 |
| ovarian cancer | 0.315 | 1.258 | 0.291 | 1.255 | 0.286 | 1.223 | 0.515 | 1.031 | 0.535 | 0.967 | 0.528 | 0.962 | 0.509 | 1.097 | 0.466 | 1.164 |
| head and neck cancer | NA | NA | NA | NA | NA | NA | 0.369 | 2.312 | 0.388 | 2.282 | 0.401 | 2.273 | 0.544 | 1.411 | 0.522 | 1.389 |
| myeloma | 0.379 | 1.401 | 0.415 | 1.397 | 0.426 | 1.138 | 0.627 | 0.937 | 0.615 | 0.923 | 0.641 | 0.913 | 0.664 | 0.771 | 0.563 | 0.873 |
| stomach cancer | 0.618 | 0.917 | 0.596 | 0.833 | 0.617 | 0.827 | 0.817 | 0.663 | 0.832 | 0.591 | 0.843 | 0.488 | 0.694 | 1.009 | 0.582 | 1.156 |
| sarcoma | 1.000 | 0.000 | 1.000 | 0.000 | 1.000 | 0.000 | 0.367 | 2.312 | 0.386 | 2.282 | 0.374 | 2.273 | 0.612 | 1.237 | 0.623 | 1.223 |
| endometrial cancer | NA | NA | NA | NA | NA | NA | 0.380 | 2.312 | 0.387 | 2.282 | 0.379 | 2.273 | 0.632 | 1.086 | 0.617 | 1.072 |
| eye cancer | 0.520 | 1.144 | 0.506 | 1.138 | 0.541 | 1.037 | 0.723 | 0.921 | 0.723 | 0.906 | 0.690 | 0.896 | 0.714 | 0.824 | 0.724 | 0.793 |
| brain cancer | 0.434 | 1.319 | 0.431 | 1.314 | 0.450 | 1.191 | 0.672 | 1.067 | 0.650 | 1.054 | 0.653 | 1.041 | 0.747 | 0.936 | 0.741 | 0.897 |
| colorectal cancer | 0.706 | 0.655 | 0.732 | 0.593 | 0.711 | 0.583 | 0.838 | 0.525 | 0.897 | 0.391 | 0.878 | 0.336 | 0.856 | 0.434 | 0.868 | 0.422 |
| skin cancer | 0.741 | 0.567 | 0.787 | 0.562 | 0.775 | 0.558 | 0.858 | 0.484 | 0.890 | 0.416 | 0.885 | 0.414 | 0.925 | 0.406 | 0.894 | 0.487 |
| pancreatic cancer | 0.596 | 0.927 | 0.627 | 0.877 | 0.619 | 0.871 | 0.797 | 0.683 | 0.786 | 0.681 | 0.814 | 0.629 | 0.885 | 0.525 | 0.881 | 0.505 |
| kidney cancer | NA | NA | NA | NA | NA | NA | NA | NA | NA | NA | 1.000 | 0.000 | 1.000 | 0.000 | 0.891 | 0.500 |
| liver cancer | NA | NA | NA | NA | NA | NA | NA | NA | NA | NA | NA | NA | NA | NA | 0.907 | 0.333 |
| prostate cancer | 1.000 | 0.000 | 1.000 | 0.000 | 1.000 | 0.000 | 1.000 | 0.000 | 0.966 | 0.125 | 0.983 | 0.125 | 0.880 | 0.488 | 0.898 | 0.479 |
| cervical cancer | NA | NA | NA | NA | NA | NA | NA | NA | NA | NA | NA | NA | 0.931 | 0.238 | 0.931 | 0.238 |
| testicular cancer | 0.862 | 0.341 | 0.862 | 0.341 | 0.858 | 0.341 | 0.919 | 0.327 | 0.929 | 0.317 | 0.931 | 0.317 | 0.942 | 0.313 | 0.945 | 0.313 |
| bladder cancer | 0.915 | 0.250 | 0.913 | 0.250 | 0.896 | 0.250 | 0.939 | 0.250 | 0.946 | 0.250 | 0.942 | 0.250 | 0.949 | 0.250 | 0.962 | 0.250 |
| esophagus cancer | 0.940 | 0.111 | 0.944 | 0.111 | 0.928 | 0.111 | 0.956 | 0.091 | 0.987 | 0.077 | 0.980 | 0.077 | 0.979 | 0.071 | 0.985 | 0.071 |
| mesothelioma | NA | NA | NA | NA | NA | NA | NA | NA | 0.966 | 0.077 | 0.973 | 0.077 | 0.979 | 0.071 | 0.984 | 0.071 |
