# Supplementary material for: Cancer-Drug Associations: A Complex System
Source: PLoS One. 2010 Apr 2;5(4):e10031. doi: 10.1371/journal.pone.0010031 (PMC2848862; doi:10.1371/journal.pone.0010031)
Supplement: Table S5 — Clinical trial numbers along with distinct drug number and specific drug number for clinical trials (0.05 MB DOC) [file pone.0010031.s021.doc]

**Table S5.** Clinical trial numbers along with distinct drug number and specific drug number for clinical trials.

| **Cancer type** | **Clinical drug trial number** | **Clinical trials distinct drug number** | **Clinical trials specific drug number** |
| --- | --- | --- | --- |
| leukemia | 170 | 37 | 2 |
| lymphoma | 121 | 42 | 3 |
| lung cancer | 121 | 30 | 0 |
| breast cancer | 97 | 37 | 2 |
| ovarian cancer | 71 | 34 | 0 |
| brain cancer | 62 | 25 | 1 |
| colorectal cancer | 61 | 16 | 0 |
| prostate cancer | 48 | 23 | 3 |
| head and neck cancer | 45 | 26 | 1 |
| mesothelioma | 40 | 8 | 0 |
| pancreatic cancer | 35 | 21 | 0 |
| skin cancer | 31 | 21 | 0 |
| kidney cancer | 30 | 27 | 0 |
| esophagus cancer | 26 | 16 | 0 |
| cervical cancer | 20 | 11 | 0 |
| liver cancer | 19 | 16 | 0 |
| stomach cancer | 19 | 13 | 0 |
| myeloma | 13 | 9 | 1 |
| testicular cancer | 11 | 14 | 0 |
| bladder cancer | 10 | 10 | 1 |
| endometrial cancer | 10 | 9 | 0 |
| sarcoma | 7 | 24 | 0 |
| eye cancer | 2 | 3 | 0 |
