# Supplementary material for: Cancer-Drug Associations: A Complex System
Source: PLoS One. 2010 Apr 2;5(4):e10031. doi: 10.1371/journal.pone.0010031 (PMC2848862; doi:10.1371/journal.pone.0010031)
Supplement: Table S6 — Cancer type pairs based on FDA drug targets, together with weight of the edges (0.17 MB DOC) [file pone.0010031.s022.doc]

**Table S6.** Cancer type pairs based on FDA drug targets, together with weight of the edges.

| **Cancer type 1** | **Cancer type 2** | **Edge weight** |
| --- | --- | --- |
| kidney cancer | liver cancer | 0.58 |
| breast cancer | lung cancer | 0.58 |
| head and neck cancer | breast cancer | 0.55 |
| head and neck cancer | colorectal cancer | 0.55 |
| colorectal cancer | lung cancer | 0.53 |
| head and neck cancer | lung cancer | 0.52 |
| colorectal cancer | breast cancer | 0.52 |
| testicular cancer | bladder cancer | 0.5 |
| sarcoma | stomach cancer | 0.5 |
| bladder cancer | eye cancer | 0.5 |
| brain cancer | eye cancer | 0.5 |
| stomach cancer | kidney cancer | 0.43 |
| head and neck cancer | lymphoma | 0.41 |
| ovarian cancer | cervical cancer | 0.38 |
| ovarian cancer | skin cancer | 0.36 |
| colorectal cancer | lymphoma | 0.34 |
| bladder cancer | brain cancer | 0.33 |
| lymphoma | breast cancer | 0.32 |
| lymphoma | lung cancer | 0.3 |
| ovarian cancer | lung cancer | 0.29 |
| bladder cancer | skin cancer | 0.29 |
| leukemia | lung cancer | 0.28 |
| leukemia | breast cancer | 0.27 |
| head and neck cancer | leukemia | 0.27 |
| sarcoma | kidney cancer | 0.26 |
| prostate cancer | skin cancer | 0.25 |
| ovarian cancer | bladder cancer | 0.25 |
| testicular cancer | eye cancer | 0.25 |
| stomach cancer | liver cancer | 0.25 |
| mesothelioma | endometrial cancer | 0.25 |
| sarcoma | leukemia | 0.24 |
| leukemia | lymphoma | 0.23 |
| testicular cancer | skin cancer | 0.22 |
| colorectal cancer | leukemia | 0.21 |
| lymphoma | myeloma | 0.21 |
| ovarian cancer | testicular cancer | 0.2 |
| testicular cancer | brain cancer | 0.2 |
| stomach cancer | leukemia | 0.19 |
| pancreatic cancer | breast cancer | 0.17 |
| sarcoma | endometrial cancer | 0.17 |
| bladder cancer | myeloma | 0.17 |
| brain cancer | myeloma | 0.17 |
| ovarian cancer | breast cancer | 0.16 |
| pancreatic cancer | colorectal cancer | 0.15 |
| prostate cancer | ovarian cancer | 0.14 |
| ovarian cancer | head and neck cancer | 0.14 |
| testicular cancer | myeloma | 0.14 |
| pancreatic cancer | mesothelioma | 0.14 |
| eye cancer | skin cancer | 0.14 |
| pancreatic cancer | lung cancer | 0.14 |
| stomach cancer | skin cancer | 0.14 |
| prostate cancer | stomach cancer | 0.13 |
| ovarian cancer | stomach cancer | 0.13 |
| ovarian cancer | colorectal cancer | 0.13 |
| skin cancer | lung cancer | 0.13 |
| ovarian cancer | eye cancer | 0.13 |
| head and neck cancer | endometrial cancer | 0.13 |
| brain cancer | skin cancer | 0.13 |
| breast cancer | skin cancer | 0.13 |
| prostate cancer | breast cancer | 0.12 |
| sarcoma | liver cancer | 0.12 |
| myeloma | skin cancer | 0.12 |
| ovarian cancer | sarcoma | 0.11 |
| ovarian cancer | brain cancer | 0.11 |
| ovarian cancer | endometrial cancer | 0.11 |
| ovarian cancer | myeloma | 0.11 |
| cervical cancer | lung cancer | 0.11 |
| mesothelioma | lung cancer | 0.11 |
| cervical cancer | colorectal cancer | 0.11 |
| testicular cancer | lung cancer | 0.1 |
| leukemia | skin cancer | 0.1 |
| pancreatic cancer | stomach cancer | 0.1 |
| head and neck cancer | skin cancer | 0.1 |
| stomach cancer | lung cancer | 0.1 |
| ovarian cancer | leukemia | 0.09 |
| stomach cancer | breast cancer | 0.09 |
| prostate cancer | head and neck cancer | 0.09 |
| kidney cancer | leukemia | 0.09 |
| eye cancer | myeloma | 0.08 |
| sarcoma | lung cancer | 0.08 |
| breast cancer | myeloma | 0.08 |
| sarcoma | head and neck cancer | 0.08 |
| sarcoma | cervical cancer | 0.07 |
| sarcoma | mesothelioma | 0.07 |
| bladder cancer | lung cancer | 0.07 |
| endometrial cancer | lung cancer | 0.07 |
| esophagus cancer | lung cancer | 0.07 |
| bladder cancer | breast cancer | 0.07 |
| brain cancer | lymphoma | 0.07 |
| endometrial cancer | lymphoma | 0.07 |
| endometrial cancer | breast cancer | 0.07 |
| mesothelioma | breast cancer | 0.07 |
| testicular cancer | breast cancer | 0.06 |
| head and neck cancer | stomach cancer | 0.06 |
| prostate cancer | lung cancer | 0.06 |
| testicular cancer | leukemia | 0.06 |
| head and neck cancer | esophagus cancer | 0.06 |
| stomach cancer | colorectal cancer | 0.06 |
| ovarian cancer | lymphoma | 0.06 |
| sarcoma | skin cancer | 0.06 |
| head and neck cancer | mesothelioma | 0.06 |
| colorectal cancer | eye cancer | 0.06 |
| prostate cancer | sarcoma | 0.05 |
| bladder cancer | colorectal cancer | 0.05 |
| brain cancer | colorectal cancer | 0.05 |
| colorectal cancer | esophagus cancer | 0.05 |
| myeloma | lung cancer | 0.05 |
| sarcoma | lymphoma | 0.05 |
| sarcoma | breast cancer | 0.05 |
| pancreatic cancer | head and neck cancer | 0.05 |
| stomach cancer | cervical cancer | 0.05 |
| stomach cancer | mesothelioma | 0.05 |
| colorectal cancer | mesothelioma | 0.05 |
| testicular cancer | colorectal cancer | 0.05 |
| colorectal cancer | skin cancer | 0.04 |
| bladder cancer | leukemia | 0.04 |
| leukemia | endometrial cancer | 0.04 |
| liver cancer | leukemia | 0.04 |
| prostate cancer | leukemia | 0.04 |
| eye cancer | lung cancer | 0.04 |
| brain cancer | lung cancer | 0.03 |
| colorectal cancer | myeloma | 0.03 |
| lymphoma | eye cancer | 0.03 |
| eye cancer | breast cancer | 0.03 |
| bladder cancer | lymphoma | 0.03 |
| brain cancer | breast cancer | 0.03 |
| leukemia | myeloma | 0.03 |
| lymphoma | esophagus cancer | 0.03 |
| esophagus cancer | breast cancer | 0.03 |
| mesothelioma | lymphoma | 0.03 |
| testicular cancer | lymphoma | 0.03 |
| lymphoma | skin cancer | 0.03 |
| leukemia | eye cancer | 0.02 |
| brain cancer | leukemia | 0.02 |
| leukemia | esophagus cancer | 0.02 |
| cervical cancer | leukemia | 0.02 |
| mesothelioma | leukemia | 0.02 |
| pancreatic cancer | leukemia | 0.02 |
