# Supplementary material for: Cancer-Drug Associations: A Complex System
Source: PLoS One. 2010 Apr 2;5(4):e10031. doi: 10.1371/journal.pone.0010031 (PMC2848862; doi:10.1371/journal.pone.0010031)
Supplement: Table S7 — Cancer type pairs based on mutation targets, together with weight of the edges (0.11 MB DOC) [file pone.0010031.s023.doc]

**Table S7.** Cancer type pairs based on mutation targets, together with weight of the edges.

| **Cancer type 1** | **Cancer type 2** | **Edge weight** |
| --- | --- | --- |
| colorectal cancer | ovarian cancer | 0.38 |
| ovarian cancer | endometrial cancer | 0.26 |
| colorectal cancer | endometrial cancer | 0.26 |
| colorectal cancer | pancreatic cancer | 0.21 |
| brain cancer | colorectal cancer | 0.19 |
| ovarian cancer | lung cancer | 0.18 |
| breast cancer | stomach cancer | 0.18 |
| colorectal cancer | breast cancer | 0.17 |
| lung cancer | breast cancer | 0.17 |
| ovarian cancer | breast cancer | 0.15 |
| colorectal cancer | lung cancer | 0.15 |
| ovarian cancer | pancreatic cancer | 0.14 |
| lung cancer | stomach cancer | 0.14 |
| pancreatic cancer | breast cancer | 0.13 |
| brain cancer | lung cancer | 0.10 |
| pancreatic cancer | lung cancer | 0.10 |
| brain cancer | ovarian cancer | 0.10 |
| colorectal cancer | liver cancer | 0.10 |
| testicular cancer | skin cancer | 0.10 |
| brain cancer | sarcoma | 0.09 |
| eye cancer | lung cancer | 0.08 |
| testicular cancer | pancreatic cancer | 0.08 |
| brain cancer | endometrial cancer | 0.08 |
| liver cancer | pancreatic cancer | 0.08 |
| endometrial cancer | stomach cancer | 0.08 |
| sarcoma | breast cancer | 0.07 |
| testicular cancer | lung cancer | 0.07 |
| brain cancer | breast cancer | 0.07 |
| myeloma | bladder cancer | 0.07 |
| thyroid cancer | pancreatic cancer | 0.06 |
| head and neck cancer | kidney cancer | 0.06 |
| eye cancer | breast cancer | 0.06 |
| ovarian cancer | testicular cancer | 0.06 |
| prostate cancer | sarcoma | 0.06 |
| thyroid cancer | lung cancer | 0.06 |
| ovarian cancer | liver cancer | 0.06 |
| ovarian cancer | stomach cancer | 0.06 |
| prostate cancer | endometrial cancer | 0.05 |
| brain cancer | prostate cancer | 0.05 |
| lymphoma | leukemia | 0.05 |
| thyroid cancer | colorectal cancer | 0.05 |
| thyroid cancer | skin cancer | 0.05 |
| endometrial cancer | lung cancer | 0.05 |
| colorectal cancer | stomach cancer | 0.05 |
| brain cancer | lymphoma | 0.04 |
| brain cancer | skin cancer | 0.04 |
| leukemia | breast cancer | 0.04 |
| sarcoma | lung cancer | 0.04 |
| pancreatic cancer | skin cancer | 0.03 |
| colorectal cancer | sarcoma | 0.03 |
| kidney cancer | breast cancer | 0.03 |
| skin cancer | lung cancer | 0.03 |
| brain cancer | bladder cancer | 0.03 |
| ovarian cancer | skin cancer | 0.03 |
| myeloma | skin cancer | 0.03 |
| brain cancer | liver cancer | 0.03 |
| brain cancer | stomach cancer | 0.03 |
| leukemia | myeloma | 0.03 |
| thyroid cancer | ovarian cancer | 0.03 |
| thyroid cancer | myeloma | 0.03 |
| lymphoma | myeloma | 0.03 |
| colorectal cancer | skin cancer | 0.03 |
| brain cancer | pancreatic cancer | 0.03 |
| lymphoma | skin cancer | 0.02 |
| leukemia | sarcoma | 0.02 |
| lymphoma | thyroid cancer | 0.02 |
| eye cancer | sarcoma | 0.02 |
| bladder cancer | sarcoma | 0.02 |
| brain cancer | kidney cancer | 0.02 |
| thyroid cancer | leukemia | 0.02 |
| lymphoma | sarcoma | 0.02 |
| brain cancer | thyroid cancer | 0.02 |
| leukemia | pancreatic cancer | 0.02 |
| kidney cancer | sarcoma | 0.02 |
| colorectal cancer | leukemia | 0.02 |
| leukemia | skin cancer | 0.02 |
| sarcoma | skin cancer | 0.02 |
| lymphoma | bladder cancer | 0.02 |
| brain cancer | leukemia | 0.02 |
