# Supplementary material for: Cancer-Drug Associations: A Complex System
Source: PLoS One. 2010 Apr 2;5(4):e10031. doi: 10.1371/journal.pone.0010031 (PMC2848862; doi:10.1371/journal.pone.0010031)
Supplement: Table S8 — Comparison of mutation target-based and drug target-based weight values of cancer pairs (0.21 MB DOC) [file pone.0010031.s024.doc]

**Table S8.** Comparison of mutation target-based and drug target-based weight values of cancer pairs.

| **Cancer type 1** | **Cancer type 2** | **Mutation target based edge weight** | **Drug target based edge weight** | **Weight difference** |
| --- | --- | --- | --- | --- |
| colorectal cancer | endometrial cancer | 0.26 | 0 | 0.26 |
| colorectal cancer | ovarian cancer | 0.38 | 0.13 | 0.24 |
| endometrial cancer | ovarian cancer | 0.26 | 0.11 | 0.15 |
| brain cancer | colorectal cancer | 0.19 | 0.05 | 0.14 |
| ovarian cancer | pancreatic cancer | 0.14 | 0 | 0.14 |
| colorectal cancer | liver cancer | 0.1 | 0 | 0.1 |
| brain cancer | sarcoma | 0.09 | 0 | 0.09 |
| brain cancer | endometrial cancer | 0.08 | 0 | 0.08 |
| breast cancer | stomach cancer | 0.18 | 0.09 | 0.08 |
| endometrial cancer | stomach cancer | 0.08 | 0 | 0.08 |
| liver cancer | pancreatic cancer | 0.08 | 0 | 0.08 |
| pancreatic cancer | testicular cancer | 0.08 | 0 | 0.08 |
| brain cancer | lung cancer | 0.1 | 0.03 | 0.07 |
| colorectal cancer | pancreatic cancer | 0.21 | 0.15 | 0.06 |
| head and neck cancer | kidney cancer | 0.06 | 0 | 0.06 |
| liver cancer | ovarian cancer | 0.06 | 0 | 0.06 |
| brain cancer | prostate cancer | 0.05 | 0 | 0.05 |
| endometrial cancer | prostate cancer | 0.05 | 0 | 0.05 |
| eye cancer | lung cancer | 0.08 | 0.04 | 0.05 |
| lung cancer | stomach cancer | 0.14 | 0.1 | 0.05 |
| brain cancer | breast cancer | 0.07 | 0.03 | 0.03 |
| brain cancer | liver cancer | 0.03 | 0 | 0.03 |
| brain cancer | pancreatic cancer | 0.03 | 0 | 0.03 |
| brain cancer | stomach cancer | 0.03 | 0 | 0.03 |
| breast cancer | eye cancer | 0.06 | 0.03 | 0.03 |
| breast cancer | kidney cancer | 0.03 | 0 | 0.03 |
| colorectal cancer | sarcoma | 0.03 | 0 | 0.03 |
| pancreatic cancer | skin cancer | 0.03 | 0 | 0.03 |
| bladder cancer | sarcoma | 0.02 | 0 | 0.02 |
| brain cancer | kidney cancer | 0.02 | 0 | 0.02 |
| breast cancer | sarcoma | 0.07 | 0.05 | 0.02 |
| eye cancer | sarcoma | 0.02 | 0 | 0.02 |
| prostate cancer | sarcoma | 0.06 | 0.05 | 0.01 |
| brain cancer | leukemia | 0.02 | 0.02 | 0 |
| breast cancer | ovarian cancer | 0.15 | 0.16 | 0 |
| leukemia | myeloma | 0.03 | 0.03 | 0 |
| leukemia | pancreatic cancer | 0.02 | 0.02 | 0 |
| lymphoma | skin cancer | 0.02 | 0.03 | 0 |
| brain cancer | ovarian cancer | 0.1 | 0.11 | -0.01 |
| colorectal cancer | stomach cancer | 0.05 | 0.06 | -0.01 |
| bladder cancer | lymphoma | 0.02 | 0.03 | -0.02 |
| colorectal cancer | skin cancer | 0.03 | 0.04 | -0.02 |
| endometrial cancer | lung cancer | 0.05 | 0.07 | -0.02 |
| eye cancer | leukemia | 0 | 0.02 | -0.02 |
| lymphoma | testicular cancer | 0.02 | 0.03 | -0.02 |
| brain cancer | lymphoma | 0.04 | 0.07 | -0.03 |
| colorectal cancer | myeloma | 0 | 0.03 | -0.03 |
| endometrial cancer | leukemia | 0.01 | 0.04 | -0.03 |
| eye cancer | lymphoma | 0 | 0.03 | -0.03 |
| leukemia | prostate cancer | 0.01 | 0.04 | -0.03 |
| lung cancer | testicular cancer | 0.07 | 0.1 | -0.03 |
| lymphoma | sarcoma | 0.02 | 0.05 | -0.03 |
| bladder cancer | leukemia | 0 | 0.04 | -0.04 |
| breast cancer | pancreatic cancer | 0.13 | 0.17 | -0.04 |
| leukemia | liver cancer | 0 | 0.04 | -0.04 |
| lung cancer | pancreatic cancer | 0.1 | 0.14 | -0.04 |
| lung cancer | sarcoma | 0.04 | 0.08 | -0.04 |
| sarcoma | skin cancer | 0.02 | 0.06 | -0.04 |
| bladder cancer | colorectal cancer | 0 | 0.05 | -0.05 |
| colorectal cancer | testicular cancer | 0 | 0.05 | -0.05 |
| head and neck cancer | pancreatic cancer | 0 | 0.05 | -0.05 |
| leukemia | testicular cancer | 0.01 | 0.06 | -0.05 |
| lung cancer | myeloma | 0 | 0.05 | -0.05 |
| breast cancer | testicular cancer | 0 | 0.06 | -0.06 |
| colorectal cancer | eye cancer | 0 | 0.06 | -0.06 |
| head and neck cancer | stomach cancer | 0 | 0.06 | -0.06 |
| lung cancer | prostate cancer | 0 | 0.06 | -0.06 |
| lymphoma | ovarian cancer | 0 | 0.06 | -0.06 |
| bladder cancer | breast cancer | 0 | 0.07 | -0.07 |
| bladder cancer | lung cancer | 0 | 0.07 | -0.07 |
| breast cancer | endometrial cancer | 0 | 0.07 | -0.07 |
| endometrial cancer | lymphoma | 0 | 0.07 | -0.07 |
| ovarian cancer | stomach cancer | 0.06 | 0.13 | -0.07 |
| brain cancer | skin cancer | 0.04 | 0.13 | -0.08 |
| breast cancer | myeloma | 0 | 0.08 | -0.08 |
| eye cancer | myeloma | 0 | 0.08 | -0.08 |
| head and neck cancer | sarcoma | 0 | 0.08 | -0.08 |
| kidney cancer | leukemia | 0.01 | 0.09 | -0.08 |
| leukemia | skin cancer | 0.02 | 0.1 | -0.08 |
| head and neck cancer | prostate cancer | 0 | 0.09 | -0.09 |
| leukemia | ovarian cancer | 0.01 | 0.09 | -0.09 |
| myeloma | skin cancer | 0.03 | 0.12 | -0.09 |
| bladder cancer | myeloma | 0.07 | 0.17 | -0.1 |
| head and neck cancer | skin cancer | 0 | 0.1 | -0.1 |
| lung cancer | ovarian cancer | 0.18 | 0.29 | -0.1 |
| lung cancer | skin cancer | 0.03 | 0.13 | -0.1 |
| pancreatic cancer | stomach cancer | 0 | 0.1 | -0.1 |
| myeloma | ovarian cancer | 0 | 0.11 | -0.11 |
| ovarian cancer | sarcoma | 0 | 0.11 | -0.11 |
| breast cancer | prostate cancer | 0 | 0.12 | -0.12 |
| liver cancer | sarcoma | 0 | 0.12 | -0.12 |
| breast cancer | skin cancer | 0 | 0.13 | -0.13 |
| endometrial cancer | head and neck cancer | 0 | 0.13 | -0.13 |
| eye cancer | ovarian cancer | 0 | 0.13 | -0.13 |
| prostate cancer | stomach cancer | 0 | 0.13 | -0.13 |
| skin cancer | testicular cancer | 0.1 | 0.22 | -0.13 |
| eye cancer | skin cancer | 0 | 0.14 | -0.14 |
| head and neck cancer | ovarian cancer | 0 | 0.14 | -0.14 |
| myeloma | testicular cancer | 0 | 0.14 | -0.14 |
| ovarian cancer | prostate cancer | 0 | 0.14 | -0.14 |
| ovarian cancer | testicular cancer | 0.06 | 0.2 | -0.14 |
| skin cancer | stomach cancer | 0 | 0.14 | -0.14 |
| brain cancer | myeloma | 0 | 0.17 | -0.17 |
| endometrial cancer | sarcoma | 0 | 0.17 | -0.17 |
| leukemia | lymphoma | 0.05 | 0.23 | -0.18 |
| lymphoma | myeloma | 0.03 | 0.21 | -0.18 |
| leukemia | stomach cancer | 0 | 0.19 | -0.19 |
| brain cancer | testicular cancer | 0 | 0.2 | -0.2 |
| colorectal cancer | leukemia | 0.02 | 0.21 | -0.2 |
| leukemia | sarcoma | 0.02 | 0.24 | -0.22 |
| breast cancer | leukemia | 0.04 | 0.27 | -0.23 |
| bladder cancer | ovarian cancer | 0 | 0.25 | -0.25 |
| eye cancer | testicular cancer | 0 | 0.25 | -0.25 |
| kidney cancer | sarcoma | 0.02 | 0.26 | -0.25 |
| liver cancer | stomach cancer | 0 | 0.25 | -0.25 |
| prostate cancer | skin cancer | 0 | 0.25 | -0.25 |
| head and neck cancer | leukemia | 0 | 0.27 | -0.27 |
| leukemia | lung cancer | 0.01 | 0.28 | -0.27 |
| lung cancer | lymphoma | 0.01 | 0.3 | -0.28 |
| bladder cancer | skin cancer | 0 | 0.29 | -0.29 |
| bladder cancer | brain cancer | 0.03 | 0.33 | -0.3 |
| breast cancer | lymphoma | 0.01 | 0.32 | -0.31 |
| ovarian cancer | skin cancer | 0.03 | 0.36 | -0.33 |
| colorectal cancer | lymphoma | 0 | 0.34 | -0.34 |
| breast cancer | colorectal cancer | 0.17 | 0.52 | -0.35 |
| colorectal cancer | lung cancer | 0.15 | 0.53 | -0.39 |
| head and neck cancer | lymphoma | 0 | 0.41 | -0.41 |
| breast cancer | lung cancer | 0.17 | 0.58 | -0.42 |
| kidney cancer | stomach cancer | 0 | 0.43 | -0.43 |
| bladder cancer | eye cancer | 0 | 0.5 | -0.5 |
| bladder cancer | testicular cancer | 0 | 0.5 | -0.5 |
| brain cancer | eye cancer | 0 | 0.5 | -0.5 |
| sarcoma | stomach cancer | 0 | 0.5 | -0.5 |
| head and neck cancer | lung cancer | 0 | 0.52 | -0.52 |
| breast cancer | head and neck cancer | 0 | 0.55 | -0.55 |
| colorectal cancer | head and neck cancer | 0 | 0.55 | -0.55 |
| kidney cancer | liver cancer | 0 | 0.58 | -0.58 |
